# Fortilin potentiates the peroxidase activity of Peroxiredoxin-1 and protects against alcohol-induced liver damage in mice

**DOI:** 10.1038/srep18701

**Published:** 2016-01-04

**Authors:** Abhijnan Chattopadhyay, Decha Pinkaew, Hung Q. Doan, Reed B. Jacob, Sunil K. Verma, Hana Friedman, Alan C. Peterson, Muge N. Kuyumcu-Martinez, Owen M. McDougal, Ken Fujise

**Affiliations:** 1 Division of Cardiology, Department of Internal Medicine, University of Texas Medical Branch at Galveston, TX 77555, USA; 2 Department of Biochemistry and Molecular Biology, University of Texas Medical Branch at Galveston, TX 77555, USA; 3 The Institute of Translational Sciences, University of Texas Medical Branch at Galveston, TX 77555, USA; 4 Department of Biochemistry and Biophysics, University of North Carolina, Chapel Hill, NC 27599, USA; 5 McGill University, Montreal, Quebec, Canada; 6 Department of Chemistry and Biochemistry, Boise State University, 1910 University Drive, Boise, ID 83725-1520, USA

## Abstract

Fortilin, a pro-survival molecule, inhibits p53-induced apoptosis by binding to the sequence-specific DNA-binding domain of the tumor suppressor protein and preventing it from transcriptionally activating Bax. Intriguingly, fortilin protects cells against ROS-induced cell death, independent of p53. The signaling pathway through which fortilin protects cells against ROS-induced cell death, however, is unknown. Here we report that fortilin physically interacts with the antioxidant enzyme peroxiredoxin-1 (PRX1), protects it from proteasome-mediated degradation, and keeps it enzymatically active by blocking its deactivating phosphorylation by Mst1, a serine/threonine kinase. At the whole animal level, the liver-specific overexpression of fortilin reduced PRX1 phosphorylation in the liver, enhanced PRX1 activity, and protected the transgenic animals against alcohol-induced, ROS-mediated, liver damage. These data suggest the presence of a novel oxidative-stress-handling pathway where the anti-p53 molecule fortilin augments the peroxidase PRX1 by protecting it against degradation and inactivation of the enzyme. Fortilin-PRX1 interaction in the liver could be clinically exploited further to prevent acute alcohol-induced liver damage in humans.

Reactive oxygen species (ROS) represent one of the most significant stress factors and threats to the wellbeing of cells and living organisms. At the whole-animal level, persistent oxidative stress has been implicated in aging^1^, neurodegenerative disorders^2^, cardiac arrhythmia^3^, osteoporosis^4^, diabetes^5^, and other conditions. When oxidative stress becomes overwhelming, the cell undergoes apoptotic death. The tumor suppressor protein p53, along with its signal transducers such as p85^6^, plays an important role in cell death induced by oxidative damage^7^. In addition, Bcl-2 and other proteins were shown to protect cells from ROS-induced cell death independently of p53^8^.

Fortilin, also known as translationally controlled tumor protein (TCTP), is a 172-amino-acid nuclear-cytosolic shuttle protein that was originally cloned in 1989 by Gross and others as a molecule abundantly expressed in tumor cells^9^. Fortilin has been implicated in various cellular functions^10^,^11^,^12^,^13^,^14^,^15^,^16^, and also possesses potent anti-apoptotic activity^11^,^17^,^18^,^19^,^20^,^21^,^22^,^23^. Fortilin binds to and stabilizes MCL1^23^, a Bcl-2 family member and macrophage survival factor^24^,^25^. In addition, fortilin binds to and destabilizes transforming growth factor-β-stimulated clone-22 (TSC-22), a pro-apoptotic protein^26^. Further, fortilin binds calcium and blocks calcium-dependent apoptosis^11^. The predominant mechanism by which fortilin blocks apoptotic cell death, however, is through its binding and inhibition of p53^27^ where fortilin binds the sequence-specific DNA-binding domain of p53 and prevents p53 from transcriptionally activating the pro-apoptotic gene Bax^27^.

Despite the well-documented anti-apoptotic activity of fortilin, its precise role in oxidative-stress-induced cell death remains unknown. We here report that fortilin protects cells against ROS-medicated apoptosis independently of p53. Fortilin does so by physically interacting with peroxiredoxin-1 (PRX1), protecting it from proteasome-mediated degradation as well as keeping it enzymatically active by shielding it from deactivating phosphorylation by mammalian sterile twenty (Mst)1^28^. At the whole animal level, fortilin collaborates with PRX1 and protects the liver against alcohol-induced, ROS-mediated, injury. We propose that fortilin-PRX1 interaction is a key mechanism by which cells cope with oxidative stress and escape apoptotic death.

## Results

### Fortilin Protects Cells against ROS-Induced Apoptosis Independently of p53

**Figure 1:**
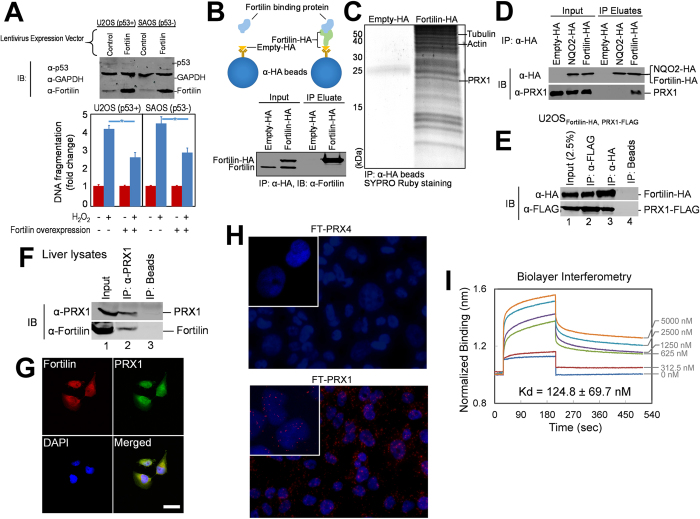
Fortilin binds PRX1. (**A**). Fortilin protects cells against H_2_O_2_-induced oxidative-damage-mediated apoptosis independently of p53. Abbreviations: IB, immunoblot; α-p53, anti-p53 antibody; α-GAPDH, anti-glyceraldehyde 3-phosphate dehydrogenase antibody; α-Fortilin, anti-fortilin antibody; *P < 0.05. Fortilin decreased DNA fragmentation in cells challenged by H_2_O_2_ regardless of the presence (U2OS) or absence (SAOS) of functional p53. (**B**). Co-immunoprecipitation system used to identify fortilin-binding proteins. HA, human influenza hemagglutinin; α-HA, anti-HA antibody; IP, immunoprecipitation. Total cell lysates from U2OS cells overexpressing HA-tagged fortilin or the HA-tag alone were subjected to immunoprecipitation by beads coated with an α-HA antibody. (**C**). Fortilin-binding proteins. PRX1, peroxiredoxin-1. Co-immunoprecipitated fortilin-binding proteins were resolved by SDS-PAGE. The bands representing fortilin-binding proteins were excised and subjected to mass spectrometry. PRX1 was identified as a new fortilin-binding protein. Known fortilin-binding proteins, such as tubulin and actin, were also co-immunoprecipitated. (**D–F**). Verification of specific interaction between fortilin and PRX1. α-PRX1, anti-peroxiredoxin antibody; NQO2, NRH-Quinone oxidoreductase-2; FLAG, FLAG (DYKDDDDK) epitope tag. Fortilin-HA, but not NQO2-HA or HA alone, co-immunoprecipitated PRX1 (**D**). Fortilin-HA co-immunoprecipitated FLAG-tagged PRX1 and vice versa (**E**). Native PRX1 co-immunoprecipitated native fortilin in cleared lysates from liver homogenates (**F**). (**G**). Immunocytochemical co-localization of fortilin and PRX1. DAPI, 4′,6-diamidino-2-phenylindole. U2OS cells were double-stained with anti-fortilin and anti-PRX1 antibodies, showing their co-localization in the perinuclear zone of the cytosol by confocal microscopy. (**H**). A proximity ligation assay (PLA) shows results that supports the interaction between fortilin and PRX1. FT-PRX4, a PLA assay using anti-PRX4 and anti-fortilin antibodies; FT-PRX1, a PLA assay using anti-PRX1 and anti-fortilin antibodies. The detection of fortilin and PRX1 indicates that they are within 30 nm of each other. The assay did not detect an interaction between fortilin and PRX4. Cells were counterstained with DAPI. (**I**). Biolayer interferometry further validates the specific interaction between fortilin and PRX1. Biotinylated fortilin was attached to the biosensor. Biolayer interferometry using various concentrations of recombinant PRX1 (0–5000 nM) shows specific binding between the two molecules at a dissociation constant of 124.8 ± 69.7 nM. See also Fig. S1.

To elucidate the role of fortilin in ROS-induced apoptosis, we stably overexpressed fortilin in U2OS and SAOS cells, osteosarcoma cell lines with and without active p53, respectively. We then challenged the cells with 500 μM of H_2_O_2_, and quantified the degree of DNA fragmentation. The overexpression of fortilin protected U2OS and SAOS cells from H_2_O_2_-induced DNA fragmentation to the same degree (Fig. 1A), suggesting that fortilin can protect cells against ROS-induced apoptosis independently of p53.

### Fortilin Physically Interacts with Peroxiredoxin-1 (PRX1)

Fortilin is not known to have peroxidase activity of its own. To explore how fortilin protects cells against ROS-induced apoptosis independently of p53, we sought fortilin-binding proteins with peroxidase activities. We first established U2OS cells overexpressing fortilin tagged with the haemagglutinin (HA)-epitope at its C-terminal end (U2OS_Fortilin-HA_). U2OS cells overexpressing only the HA-tag (U2OS_Empty-HA_) were used as a control throughout the experiment. We then optimized parameters for co-immunoprecipitation where fortilin-HA is immunoprecpitated by anti-HA-coated agarose beads, co-immunoprecipitating fortilin-interacting proteins with it (Fig. 1B). Total cell lysates (10 mg each) from U2OS_Empty-HA_ and U2OS_Fortilin-HA_ were subjected to the above co-immunoprecipitation strategy. Proteins co-immunoprecipitated with empty-HA (control) or fortilin-HA were resolved with 12% SDS-PAGE and stained with SYPRO Ruby. Although empty-HA failed to co-immunoprecipitate any proteins detectable with SYPRO Ruby staining, fortilin-HA co-immunoprecipitated a number of proteins detectable as discrete bands (Fig. 1C), which we immediately excised from the gel and subjected to mass-spectrometric analyses. We detected known fortilin-interacting proteins, including actin (Fig. 1C-Actin)^29^ and tubulin (Fig. 1C-Tubulin)^30^. In addition, a protein from a ~22-kDa band was identified as PRX1 (Fig. 1C-PRX1). PRX1, a 199-amino-acid polypeptide, is a member of the peroxiredoxin family (PRXs1-6; Fig. S1A) and may exist as an α_2_-homodimer or a doughnut-shaped (α_2_)_5_ homodecamer^31^. The peroxiredoxins exert their antioxidant role in cells through their peroxidase activity by reducing and detoxifying H_2_O_2_, peroxynitrite, and other organic hydroperoxides (ROOH; ROOH + 2e^−^ → ROH + H_2_O)^32^. Their peroxidase activity is driven by the oxidation of an active-site cysteine (the peroxidatic cysteine) to a sulfenic acid by the peroxide substrate. PRX1, like PRX2–5 (but unlike PRX6 which contains only one cysteine residue), contains two peroxidatic cysteine residues (Fig. S1A, Cys^52^ and Cys^173^)^32^,^33^,^34^. We examined the expression of PRX1 in various tissues by using RT-qPCR and found that it was most abundantly expressed in the kidney, liver, and lung (Fig. S1B).

We then verified the interaction between fortilin and PRX1 by performing cell-based co-immunoprecipitation assays using U2OS_Empty-HA_, U2OS_NQO2-HA_, and U2OS_Fortilin-HA_ cells. The presence of co-immunoprecipitated PRX1 was evaluated with an anti-PRX1 antibody (Fig. 1D). The anti-PRX1 antibody used in the current study, a goat polyclonal antibody raised against a synthetic peptide of PRX1, S^106^DPKRTIAQDYG^117^, is specific to PRX1, and had little or no cross reactivity with other PRXs (Fig. S1C). NQO2, NAD(P)H:quinone oxidoreductase 2, is a 231 amino acid (26 kDa) redox protein, the size and function of which are similar to those of PRX1^35^,^36^. Fortilin, but not NQO2 or HA-alone, co-immunoprecipitated PRX1 (Fig. 1D). We then generated a U2OS cell line that stably co-expressed fortilin-HA and PRX1-FLAG (U2OS_Fortilin-HA, PRX1-FLAG_) (Fig. 1E, lane 1). The total lysate from the cells was divided into three equal portions for immunoprecipitation with an anti-FLAG antibody, anti-HA antibody, or bare beads. The anti-FLAG antibody co-immunoprecipitated fortilin-HA along with PRX1-FLAG (Fig. 1E, lane 2). Conversely, anti-HA antibody successfully co-immunoprecipitated PRX1-FLAG along with fortilin-HA (Fig. 1E, lane 3). Neither fortilin-HA nor PRX1-FLAG was immunoprecipitated or co-immunoprecipitated by the bare beads (Fig. 1E, lane 4). Finally, we subjected a mouse liver lysate to immunoprecipitation with anti-PRX1 or bare beads. Anti-PRX1, but not bare beads, immunoprecipitated PRX1 (Fig. 1F, lanes 2 and 3, top panel) and co-immunoprecipitated fortilin (Fig. 1F, lane 2 and 3, bottom panel). These findings suggest the presence of a specific interaction between fortilin and PRX1.

To evaluate the spatial localization of fortilin in relation to PRX1, U2OS cells were immunostained with mouse anti-fortilin and goat anti-PRX1 antibodies. Bound antibodies were detected by donkey anti-mouse Alexa Fluor® 488 and donkey anti-goat AlexaFluor® 568 (Invitrogen, Grand Island, NY) secondary antibodies, respectively. The distribution of the two proteins was similar with the highest amount in the peri-nuclear area of the cytosol, suggesting that fortilin interacts with PRX1 in this region (Fig. 1G). To further validate the presence of a fortilin-PRX1 interaction *in situ*, we performed a proximity ligation assay (PLA)^37^ in wild-type U2OS cells, using mouse anti-fortilin and goat anti-PRX1 and anti-PRX4 antibodies. U2OS cells abundantly express both PRX1 and PRX4 (Fig. S1D) but fortilin did not interact with PRX4 in co-immunoprecipitation assays (Fig. S1E). The red dots in Fig. 1H indicate that fortilin was within about 30 nm of PRX1 in numerous locations in the cells whereas there were no signals seen for fortilin and PRX4, suggesting that they were not within close proximity. To further characterize the interaction between fortilin and PRX1, we subjected recombinant human fortilin and PRX1 (Fig. S1F) to biolayer interferometry (BLItz, ForteBio, Menlo Park CA). Results from three independent experiments indicated that fortilin specifically bound to PRX1 with a Kd of 124.8 ± 69.7 nM (Fig. 1I).

### Fortilin Protects PRX1 Against Proteasome-Mediated Degradation in Cultured Cells

**Figure 2:**
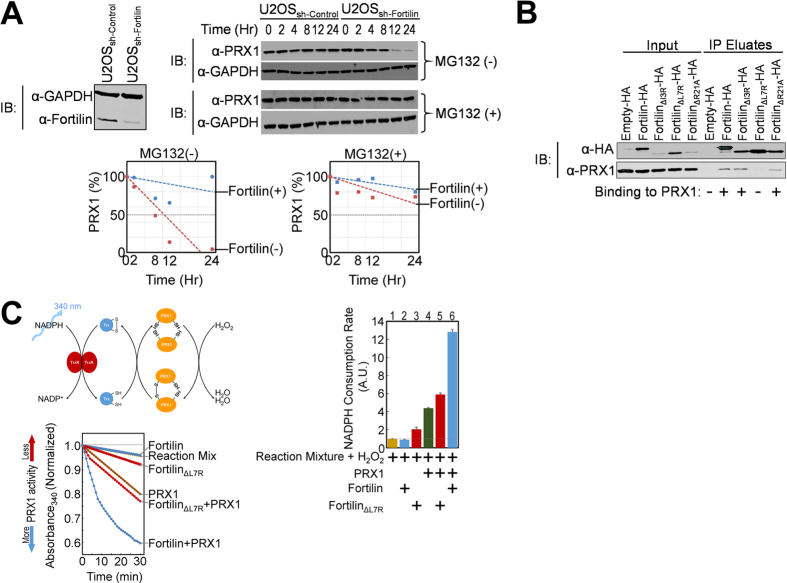
Biological significance of the fortilin-PRX1 interaction. (**A**). Fortilin protects PRX1 from proteasome-mediated degradation. Abbreviations: IB, immunoblot; α-GAPDH, anti-GAPDH antibody; α-fortilin, anti-fortilin antibody; α-PRX1, anti-peroxiredoxin-1 antibody; U2OS_sh-Control_, U2OS cells stably transfected with empty shRNA lentiviral vector; U2OS_sh-Fortilin_, U2OS cells stably transfected with anti-fortilin shRNA lentiviral vector. PRX1 was rapidly degraded through the proteasome pathway in the absence of fortilin, but this degradation was blocked by the proteasome inhibitor MG132. (**B**). Identification of a point mutant of fortilin lacking PRX1 binding. α-HA, anti-human influenza hemagglutinin antibody. A fortilin point mutant with the 7^th^ leucine (L) mutated to arginine (R) (fortilin_ΔL7R_) failed to bind PRX1 in a co-immunoprecipitation-Western blot analysis. Fortilin-HA, but not other HA-tagged fortilin mutants, contains an additional epitope tag and migrates more slowly on the SDS gel than do the fortilin mutants. (**C**). Fortilin binds PRX1 and augments PRX1 activity. NADPH, nicotinamide adenine dinucleotide phosphate; TrxR, thioredoxin reductase; Trx, thioredoxin; A.U., arbitrary unit. Fortilin, but not fortilin_ΔL7R_ that lacks PRX1 binding, enhanced PRX1 activity *in vitro*. Consumption of nicotinamide adenine dinucleotide phosphate (NADPH) was used as an indicator of PRX1 activity. Results are shown as the mean ± SD from three independent experiments. See also Fig. S2

Fortilin is known to interact with MCL1 and to protect MCL1 from proteasome-mediated degradation^38^. To test if fortilin also protects PRX1 against proteasome-mediated degradation, we used a cell line in which fortilin expression is suppressed by a small-hairpin RNA against fortilin (shRNA_fortilin_) as described by us previously^27^. U2OS_sh-Fortilin_ cells expressed much less fortilin than did U2OS_sh-Control_ cells (Fig. 2A). We treated these cells with cycloheximide, incubated them in the presence or absence of the proteasome inhibitor MG132, harvested them at various time points, and subjected their lysates to Western blot analysis to quantify the status of PRX1 within the cells. Without MG132, PRX1 disappeared more quickly from the cells in the absence of fortilin (U2OS_sh-Fortilin_) than in its presence (U2OS_sh-Control_). Strikingly, however, the difference was no longer evident in the presence of MG132 (Fig. 2A), making it unlikely that the PRX1 is degraded by processes other than proteasome-mediated pathways (such as autophagy pathways). The mRNA levels of PRX1 did not differ between U2OS_sh-Fortilin_ and U2OS_sh-Control_ cells (Fig. S2A). Lentiviral overexpression of fortilin did not change the expression levels of PRX1 in U2OS cells (Fig. S2C). Strikingly, however, lentiviral overexpression of fortilin decreased the poly-ubiquitinated PRX1s in U2OS cells (Fig. S2D). These data, when taken together, suggest that fortilin binds PRX1 and protects it from proteasome-mediated degradation.

### Fortilin Binds to PRX1 and Augments Its Peroxidase Activity *In Vitro*

Next, we screened fortilin point mutants for their ability to interact with PRX1. We transfected U2OS cells with mammalian expression plasmids containing HA-tagged fortilin or its point mutants and subjected lysates of the cells to co-immunoprecipitation and Western blot analysis. Fortilin, fortilin_ΔI3R_, and fortilin_ΔR21A_, but not fortilin_ΔL7R_, bound PRX1 (Fig. 2B). We then generated recombinant fortilin and fortilin_ΔL7R_, from 239T cells overexpressing the fortilins tagged with Strep-tag®, using the Strep-Tactin® affinity purification system^39^ (Fig. S2B). We evaluated the impact of fortilin and fortilin_ΔL7R_ on the peroxidase activity of PRX1 with the *in vitro* method described by Kim *et al.*^40^ PRX1 activity, which is measured as the nicotinamide adenine dinucleotide phosphate (NADPH) consumption rate, was normalized to 1.00 at baseline (Fig. 2C, “Reaction Mix” in the time course graph and lane 1 in the NADPH Consumption Rate graph). The addition of fortilin did not substantively increase the NADPH consumption rate, confirming that fortilin does not have significant endogenous peroxidase activity in and of itself (0.89, Fig. 2C, “Fortilin” and lane 2). As expected, the addition of PRX1 increased the NADPH consumption rate from 1.00 to 4.38 (Fig. 2C, “PRX1” and lane 4). In this system, the addition of fortilin to PRX1 further increased its peroxidase activity from 4.38 to 12.82, a 2.9-fold increase (Fig. 2C, “Fortilin + PRX1” and lane 6). Strikingly, the addition of fortilin_ΔL7R_, which does not bind PRX1, only modestly increased PRX1′s peroxidase activity from 4.38 to 5.90, a 1.3-fold increase (Fig. 2C, “Fortilin_ΔL7R_ + PRX1” and lane 5). Taken together, these data suggest that fortilin binds PRX1 and enhances its peroxidase activity. The binding of fortilin to PRX1 is required for the full augmentation of PRX1′s peroxidase activity.

### Fortilin Protects PRX1 from Phosphorylation by Mst1

**Figure 3:**
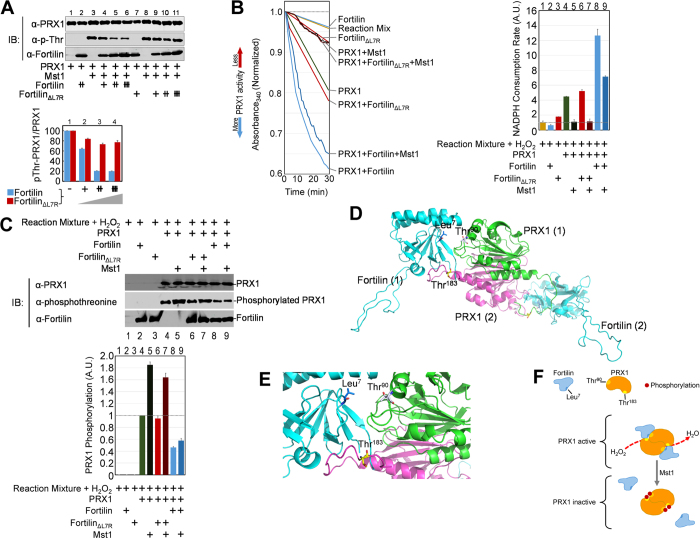
Fortilin prevents Mst1 from phosphorylating and deactivating PRX1. (**A**). An *in vitro* phosphorylation assay shows the binding-dependent inhibitory effect of fortilin on PRX1 phosphorylation by Mst1. Abbreviations: IB, immunoblot; α-PRX1, anti-PRX1 antibody; α-p-Thr, anti-phosphothreonine antibody; α-Fortilin, anti-fortilin antibody. Increasing doses of recombinant human fortilin (lanes 3–6), but not its mutant (fortilin_ΔL7R_, lanes 8–11), decreased phosphorylation of PRX1 by Mst1 *in vitro*. Densitometry was used to quantify the amount of threonine-phosphorylated PRX1. (**B**). Fortilin, but not fortilin_ΔL7R_, protects PRX1 enzymatic activity from inhibition by Mst1. NADPH, nicotinamide adenine dinucleotide phosphate; A.U., arbitrary unit. Results are shown as the mean ± SD three independent experiments. (**C**). Fortilin preserves the enzymatic activity of PRX1 by preventing Mst1 from phosphorylating PRX1. IB, immunoblot; α-phosphothreonine, anti-phosphothreonine antibody; α-Fortilin, anti-fortilin antibody; A.U., arbitrary unit derived from the densitometric ratio of the phosphorylated PRX1 threonine band to the respective total PRX1 band. The means and errors ( ± SD) of the graph were calculated from three independent experiments. (**D**). The PRX1 dimer interacts with two fortilin molecules. Fortilin occludes the PRX1 Thr^183^ phosphorylation site on the C-terminal tail of one subunit of the dimer and Thr^90^ on the second subunit. (**E**). Interaction facet between fortilin and dimerized PRX1s. (**F**). A model of physical and functional interaction between dimerized PRX1s and fortilins. Without fortilin, PRX1 is accessible by Mst1 for the phosphorylation of Thr^90^ and Thr^183^, key activity-regulating residues of PRX1. See also Fig. S3.

PRX1 is inactivated when its threonine residues (Thr^90^ and Thr^183^) are phosphorylated by Mst1^28^. To test whether Mst1 is capable of phosphorylating PRX1 in the presence of fortilin, we incubated recombinant human Mst1 with PRX1 and various amounts of fortilin or fortilin_ΔL7R_ and subjected the reaction mixture to Western blot analysis using an anti-phosphothreonine antibody. Wild-type fortilin (Fig. 3A, lanes 3–6 on the top panel), but not fortilin_ΔL7R_ that lacks PRX1 binding (Fig. 3A, lanes 8–11), blocked threonine phosphorylation of PRX1 in a dose-dependent manner (Fig. 3A, pThr-PRX1/PRX1 on the bottom panel).

Next, we evaluated the effect of Mst1 on PRX1 enzymatic activity, as measured by the NADPH consumption rate, in the presence and absence of wild-type fortilin and fortilin_ΔL7R_. In this assay, Mst1 decreased PRX1 enzymatic activity by 75.3% (PRX1 vs. PRX1 + Mst1 = 4.49 vs. 1.11 [A.U.]; Fig. 3B; the left panel as well as the lanes 4 vs. 5 of the right panel). Fortilin, but not fortilin_ΔL7R,_ robustly prevented the decrease in PRX1 enzymatic activity by Mst1 (PRX1 + fortilin_ΔL7R_ vs. PRX1 + fortilin_ΔL7R_ + Mst1 = 5.21 vs. 1.15 [A.U.], 77.9% decrease; PRX1 + fortilin vs. PRX1 + fortilin + Mst1 = 12.65 vs. 7.15 [A.U.], 43.5% decrease; Fig. 3B; the left panel and the lanes 6 vs. 7 and lanes 8 vs. 9 of the right panel). We then subjected the reaction mixtures above to Western blot analysis to evaluate the status of threonine phosphorylation of PRX1. Mst1 increased the phosphorylation of the PRX1 threonine residues (Fig. 3C, lanes 4 vs. 5 of both top and bottom panels). Wild-type fortilin (Fig. 3C, lanes 8 vs. 9), but not fortilin_ΔL7R_ (Fig. 3C, lanes 6 vs. 7), prevented Mst1 from phosphorylating PRX1. The presence of phosphorylated PRX1 in the absence of Mst1 in Fig. 3C (lanes 4, 6, and 8) is most likely due to the presence of phosphorylating proteins in the reaction mixture.

### Fortilin Binding to the PRX1 Dimer Obliterates the PRX1 phosphorylation sites

To evaluate how fortilin prevents Mst1 from phosphorylating the two key threonine residues (Thr^90^ and Thr^183^) and keeps PRX1 unphosphorylated and active, we performed computational modeling of fortilin (PDB ID: 2HR9)^41^ and PRX1 (PDB ID: 2RII)^42^ in DockoMatic V. 2.0^43^. Initial docking experiments between the two proteins were configured such that Leu^7^ on fortilin was within the ligand-binding domain, established using the grid parameter file application in AutoDock Tools^44^. Rigid docking of the PRX1 monomer to fortilin demonstrated favorable interaction with Leu^7^ of fortilin in a cleft near the center of the protein-protein interface. With only the PRX1 monomer docked, Thr^90^ was observed to be in contact with fortilin, thus rendering it inaccessible to phosphorylation by various kinases including Mst1 and cyclin-dependent kinases (CDKs) such as Cdc2^28^,^45^. Flexible docking of the C-terminal tail of a second PRX1 monomer was then performed across the entire surface of the fortilin protein. The fortilin-PRX1 C-terminal tail complex forms a favorable cluster that shields Thr^183^ from phosphorylation by Mst 1 kinases. The preferred orientation of the PRX1 C-terminal tail was then used as a guide to introduce the PRX1 dimer^46^. Figure 3D shows the interaction between the two fortilin and two PRX1 proteins. The interaction between dimerized PRX1s and a fortilin molecule is depicted in Fig. 3E and Fig. S3. These findings support the hypothesis that binding of fortilin to PRX1 prevents kinases from accessing the phosphorylation sites, but only when PRX1 is complexed with a second PRX1 protein. The backside of the PRX1 dimer is accessible to binding to a second equivalent of fortilin to prevent phosphorylation of Thr^90^ on the top PRX1 and Thr^183^ on the bottom PRX1. Taken together, these data suggest that fortilin, by binding dimerized PRX1s, protects Thr^90^ and Thr^183^ against phosphorylation by Mst1, thereby allowing the enzyme to maintain its peroxidase activity (Fig. 3F).

### Fortilin Overexpression in Mice Protects the Liver from Alcohol-Induced ROS-Mediated Damage

To explore the clinical relevance of the fortilin-PRX1 interaction in intact animals, we generated a line of mice overexpressing fortilin specifically in the liver. To this end, we generated a targeting cassette by placing the conditional stop-lox cassette^47^ upstream of the mouse fortilin cDNA sequence, thus preventing expression of the fortilin transgene in the absence of the Cre-transgene (Fig. S4A). The targeting cassette was then introduced as a single-copy transgene to the hypoxanthine phosphoribosyltransferase (HPRT) locus using homologous recombination in ES cells as we previously described^48^.

**Figure 4:**
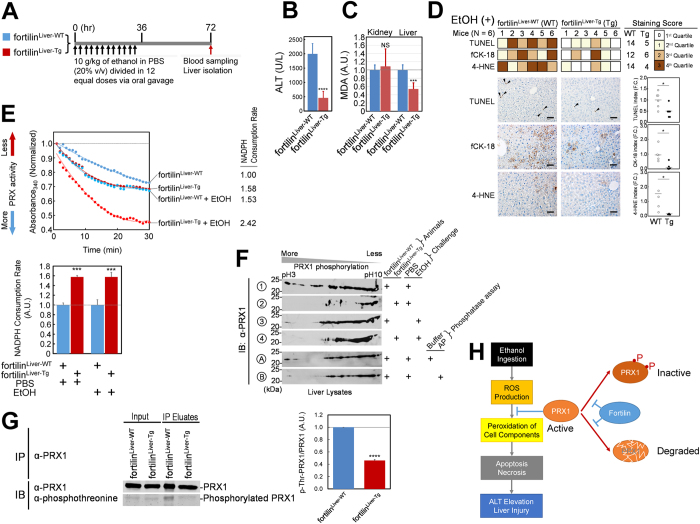
Fortilin protects against alcohol-induced ROS-mediated liver damage by augmenting PRX1 activity. (**A**). Induction of alcohol-induced liver damage in mice. A total of 10 g/kg of alcohol—dissolved in PBS— was administered via oral gavage every 3 hours in 12 equally divided doses. (**B**). Fortilin protects against alcohol-induced liver damage. Abbreviations: ALT, alanine aminotransferase. ****P < 0.001. (**C**). Fortilin protects the liver against alcohol-induced oxidative damage. MDA, malondialdehyde; A.U., arbitrary unit; NS, not significant. ***P < 0.005. The level of MDA, an indicator of oxidative tissue damage, is significantly less in the livers of fortilin^Liver-Tg^ mice than in the livers of fortilin^Liver-WT^ mice. (**D**). Fortilin reduces apoptosis and oxidative damage in alcohol-challenged mouse liver. TUNEL, terminal deoxynucleotidyl transferase dUTP nick end labeling; fCK-18, fragmented cytokeratin-18; 4-HNE, 4-hydroxy nonenal; NS, not statistically significant. Size bar = 100 μm. *P < 0.05. Semiquantitative scoring, representative histochemistry, and quantitative measurements are shown. (**E**). Fortilin augments peroxiredoxin activity in the liver. NADPH, nicotinamide adenine dinucleotide phosphate; EtOH, ethanol. ***P < 0.005 (N = 3). A peroxiredoxin assay (measured as consumption of NADPH) was performed on cleared lysates of the livers from PBS- or alcohol-challenged fortilin^Liver-WT^ and fortilin^Liver-Tg^ mice. (**F**). Fortilin decreases phosphorylation of PRX1 in the liver. IB, immunoblot; α-PRX1, anti-PRX1 antibody; AP, alkaline phosphatase. 1–4: Two-dimensional (2D) gel electrophoresis of lysates from the liver of wild-type and fortilin-transgenic mice challenged by either PBS or ethanol (EtOH). A&B: Lysates from the liver of wild-type mice challenged by PBS were treated with buffer alone (A) or AP (B) to evaluate which PRX1 signals represented phosphorylated PRX1. Isoelectric focusing was performed from pH 10 to pH 3. (**G**). Fortilin protects against deactivating threonine phosphorylation of PRX1 in the liver. IP, immunoprecipitation; α-phosphothreonine, anti-phosphothreonine antibody; p-Thre-PRX1, threonine-phorphorylated PRX1. ****P < 0.001 (N = 3). PRX1 was immunoprecipitated from the total lysates of fortilin^Liver-WT^ and fortilin^Liver-Tg^ livers. The amount of threonine-phosphorylated (and inactive) PRX1 was assessed by quantitative Western blot analyses. (**H**). Fortilin blocks the phosphorylation and degradation of PRX1. P, phosphorylation to threonine residue; ROS, reactive oxygen species; ALT, alanine aminotransferase. See also Fig. S4.

Because the HPRT locus exists on the X-chromosome, male and female mice could maximally have one and two construct copies, respectively, knocked into the locus. These transgenic knock-in mice were referred to as fortilin^Tg/Tg^ or fortilin^Tg/WT^ in females, and fortilin^Tg/−^ or fortilin^WT/−^ in males. To avoid the issue of X-inactivation of the female HPRT locus, we exclusively used male mice in the current work. We crossed these animals with a line of C57BL/6J mice overexpressing the Cre-transgene from the liver-specific albumin promoter (Alb-Cre^+/+^ mice, The Jackson Laboratories). We found that fortilin transgene expression was substantively higher in Alb-Cre^+/+^ fortilin^Tg/−^ than in Alb-Cre^+/−^fortilin^Tg/−^ mice, suggesting that high expression of Cre-recombinase is required for the LoxP-neo-stop-LoxP sequence (Fig. S4B) to be removed most efficiently. We therefore performed all subsequent experiments using male Alb-Cre^+/+^ fortilin^Tg/−^ (denoted fortilin^Liver−Tg^ hereafter) and Alb-Cre^+/+^ fortilin^WT/−^ (denoted fortilin^Liver-WT^ hereafter) mice (Fig. S4A). The liver of fortilin^Liver-Tg^ (Alb-Cre^+/+^ fortilin^Tg/−^) mice expressed 35.5-fold and 2.4-fold more mRNA and protein than Alb-Cre^−/−^fortilin^Tg/−^ or Alb-Cre^+/−^fortilin^Tg/−^ mice, respectively (Fig. S4B). PRX1 is the most active of the 2-Cys PRXs in eliminating ROS from the livers of alcohol-challenged mice, and it protects the liver against alcohol-induced oxidative injury^49^. Because fortilin augments the peroxidase activity of PRX1 *in vitro* (Fig. 2C), we tested whether overexpression of fortilin in the liver protects the liver against alcohol-induced, oxidative-stress-mediated injury. Alcohol, not other ROS-producing agents such as H_2_O_2_ or liver ischemia-reperfusion model^50^, was used to challenge the liver with oxidative stress, because of its clinical and translational relevancy^51^,^52^. We administered 10 g/kg body weight of alcohol to fortilin^Liver-WT^ and fortilin^Liver-Tg^ mice (N = 6 per group) over 36 hours to induce acute alcohol-induced liver injury (Fig. 4A). Although all animals survived the alcohol challenge, the alanine aminotransferase (ALT) levels of wild-type mice (fortilin^Liver-WT^) increased to 1997 ± 362.5 (IU/L), 4.3-fold the level in mice overexpressing fortilin in the liver (460 ± 226.3 [IU/L], fortilin^Liver-Tg^)(Fig. 4B). The ALT levels of the two groups were similar and low at base line before administration of alcohol (fortilin^Liver-WT^ vs. fortilin^Liver-Tg^ = 36.3 ± 11.0 vs. 32.7 ± 8.1 [IU/L], NS, Fig. S4C). To test whether overexpressed fortilin protected the liver against alcohol-induced lipid peroxidation, we measured malondialdehyde (MDA) levels in the kidney (control) and liver as described previously^53^. We found that the MDA level was significantly lower in the livers, but not in the kidneys, of fortilin^Liver-Tg^ mice than of fortilin^Liver-WT^ mice (fortilin^Liver-WT^ vs. fortilin^Liver-Tg^ = 1.0 ± 0.12 vs. 0.54 ± 0.15 [fold change (F.C.)], *P* < 0.05, for the liver) (Fig. 4C). The MDA levels of the two groups were similar and low at base line before administration of alcohol (fortilin^Liver-WT^ vs. fortilin^Liver-Tg^ = 0.14 ± 0.03 vs. 0.12 ± 0.06 [A.U.] for the kidney and 0.11 ± 0.01 vs. 0.08 ± 0.04 [A.U.] for the liver, NS for both organs, Fig. S4D). The level of 4-hydroxynonenal (4-HNE), another indicator of lipid peroxidation, was also significantly lower in the livers of fortilin^Liver-Tg^ mice than in the livers of fortilin^Liver-WT^ mice (fortilin^Liver-WT^ vs. fortilin^Liver-Tg^ = 1.0 ± 0.65 vs. 0.13 ± 0.03 [F.C.], *P* < 0.05) (Fig. 4D, 4-HNE). Hepatocytes underwent significantly more apoptosis in fortilin^Liver-WT^ mice than they did in fortilin^Liver-Tg^ mice as assessed by terminal deoxynucleotidyl transferase dUTP nick end labeling (TUNEL) and fragmented cytokeratin-18 (fCK-18)^54^ indices (TUNEL index: fortilin^Liver-WT^ vs. fortilin^Liver-Tg^ = 1.0 ± 0.35 vs. 0.48 ± 0.26 [F.C.], *P* < 0.05; fCK-18 index: fortilin^Liver-WT^ vs. fortilin^Liver-Tg^ = 1.0 ± 0.51 vs. 0.25 ± 0.25 [F.C.], *P* < 0.05)(Fig. 4D, TUNEL and fCK-18). These observations were also overall consistent with the outcome of semi-quantitative grading of the 3,3′-diaminobenzidine (DAB) signal for TUNEL, fCK-18, and 4-HNE, showing that the liver of fortilin^Liver-WT^ mice (WT) exhibited more extensive TUNEL, fCK-18, and 4-HNE staining than that of fortilin^Liver-Tg^ mice (TG) (Fig. 4D, Staining Score: WT vs. TG = 14 vs. 5 for TUNEL; 12 vs. 6 for fCK-18; 14 vs. 4 for 4-HNE). Without alcohol treatment, there was no statistically significant difference in TUNEL, fCK-18, or 4-HNE staining between fortilin^Liver-WT^ (WT) fortilin^Liver-Tg^ (TG) mice (Fig. S4E).

To test whether PRX1 was more enzymatically active in the livers of fortilin^Liver-Tg^ mice than in those of fortilin^Liver-WT^ mice, we subjected liver lysates from these animals, which were challenged by either PBS or ethanol, to the PRX1 activity assay as described for Fig. 2C. Because the liver expresses multiple PRXs and the substrate used in the assay was not specific for PRX1, the decline in absorbance at 340 nm represented the total PRX activities, not PRX1 activity per se. The amounts of PRX1 protein expressed in the livers of fortilin^Liver-WT^ and fortilin^Liver-Tg^ mice were comparable in the absence of EtOH challenge (Fig. S4F, “PRX1” in lanes 1–3 vs. lanes 4–6). In this system, in the absence of the EtOH challenge, the lysates from fortilin^Liver-Tg^ mice exhibited 58% more PRX activity than lysates from fortilin^Liver-WT^ mice (fortilin^Liver-WT^ vs. fortilin^Liver-Tg^ mice = 1.00 ± 0.04 vs. 1.58 ± 0.03 [A.U.], *P* < 0.005, Fig. 4E, both the top line chart and bottom bar graph). Upon the EtOH challenge, PRX1 was robustly induced in the livers of fortilin^Liver-WT^ and fortilin^Liver-Tg^ mice (Fig. S4F, “PRX1”, lanes 1–3 vs. lanes 7–9 for fortilin^Liver-WT^ mice; lanes 4–6 vs. lanes 10–12 for fortilin^Liver-WT^ mice). With EtOH challenge, the PRX1 protein levels were higher in the liver of fortilin^Liver-Tg^ than that of fortilin^Liver-WT^ mice (Fig. S4F, “PRX1” in lanes 7–9 vs. lanes 10–12), which might be due to the protection by fortilin of PRX1 against ubiquitination (Fig. S4G, lanes 3 vs. 4) and proteasome-mediated degradation (Fig. 2A). In this system, in the presence of the EtOH challenge, the lysates from fortilin^Liver-Tg^ mice exhibited 58% (the same as in the absence of the EtOH challenge) more PRX activity in the liver than lysates from fortilin^Liver-WT^ mice (fortilin^Liver-WT^ vs. fortilin^Liver-Tg^ mice = 1.00 ± 0.11 vs. 1.58 ± 0.09 [A.U.], *P* < 0.005, Fig. 4E, both the top line chart and bottom bar graph). Highest PRX1 enzymatic activities were seen in the liver of EtOH-challenged fortilin^Liver-Tg^ mice (2.42 ± 0.12 [A.U.]), followed equally by EtOH-challenged fortilin^Liver-WT^ mice (1.58 ± 0.03 [A.U.]) and PBS-treated fortilin^Liver-Tg^ mice (1.53 ± 0.14 [A.U.]), and then by PBS-treated fortilin^Liver-WT^ mice (1.00 ± 0.04 [A.U.])(Fig. 4E). These data suggest that PRX1 is more enzymatically active in the livers of fortilin^Liver-Tg^ mice than in those of fortilin^Liver-WT^ mice, regardless of EtOH challenge.

Next, we tested whether PRX1 was better protected from deactivating phosphorylation in the liver of fortilin^Liver-Tg^ mice than in the liver of fortilin^Liver-WT^ mice. We evaluated the status of PRX1 phosphorylation by using 2-dimensional (2D) gels followed by Western blotting in liver lysates from fortilin^Liver-WT^ and fortilin^Liver-Tg^ mice which were challenged either by PBS or EtOH (N = 3 per group). Regardless of EtOH challenge, all of the fortilin^Liver-WT^ tissues examined expressed highly acidic isoforms of PRX1 in comparison to the liver tissues from fortilin^Liver-Tg^ mice (Fig. 4F, signals seen close to “pH3” of the 2D gels; panels 1 vs. 2 for PBS; panels 3 vs. 4 for EtOH). In addition, both fortilin^Liver-WT^ and fortilin^Liver-Tg^ tissues expressed less highly acidic isoforms of PRX1 when challenged by EtOH than they did when challenged by PBS (Fig. 4F, PBS vs. EtOH: panels 1 vs. 3 for fortilin^Liver-WT^ mice; panels 2 vs. 4 for fortilin^Liver-Tg^ mice), suggesting that EtOH induces PRX1 dephosphorylation in both fortilin^Liver-WT^ and fortilin^Liver-Tg^ liver as previously shown for other liver proteins^55^.

To determine whether the acidic spots that were absent in fortilin^Liver-Tg^ mice were phosphorylated species of PRX1, we treated fortilin^Liver-WT^ lysates that displayed highly acidic spots with calf intestinal alkaline phosphatase (AP) or buffer alone (Fig. 4F, panels A,B). Buffer treatment led to disappearance of a few of the acidic spots, likely due to endogenous phosphatase activity (Fig. 4F panels 1 vs. A). The majority of the highly acidic spots in fortilin^Liver-WT^ were abolished or shifted to a basic pH after AP treatment, producing a pattern resembling that in fortilin^Liver-Tg^ livers (Fig. 4F, panel 2 vs. B). Ponceau S staining revealed similar amounts of proteins on the membranes used for the Western blots (Fig. S4H).

These data suggest that fortilin negatively regulates PRX1 phosphorylation in the mouse liver, regardless of EtOH challenge.

To more directly evaluate the status of threonine phosphorylation of PRX1 in the liver, we immunoprecipitated PRX1 from the cleared liver lysates of fortilin^Liver-WT^ and fortilin^Liver-Tg^ mice, using anti-PRX1 antibody conjugated to Protein A/G agarose beads. Immunoprecipitated PRX1 was subjected to SDS-PAGE and Western blot analysis using anti-PRX1 and anti-phosphothreonine antibodies. There were equal amounts of PRX1 in the lysates from fortilin^Liver-WT^ and fortilin^Liver-Tg^ mice (Fig. 4G, Input). Approximately same amounts of PRX1 were immunprecipitated from the lysates from fortilin^Liver-WT^ and fortilin^Liver-Tg^ mice (Fig. 4G, IP Eluates, PRX1). Strikingly, in this system, PRX1 from fortilin^Liver-WT^ mice was more extensively phosphorylated at threonine residues than that from fortilin^Liver-Tg^ mice (Fig. 4G, Phosphorylated PRX1 in the right panel and the graph in the left).

These animal data, taken together with the cellular and *in vitro* data described above, suggest that fortilin increases PRX1 enzymatic activities in the liver by both (a) protecting PRX1 against de-activating phosphorylation (Figs 4F,G, 3A,C) and (b) increasing PRX1 levels by inhibiting ubiquitination and proteasome-mediated degradation of PRX1 (Figs S4G, 2A, & S2D). Without EtOH, the former mechanism ([a]) likely plays a major role in the increase in PRX1 activity (Fig. 4E) because PRX1 protein levels were equal in fortilin^Liver-WT^ and fortilin^Liver-Tg^ mice (Fig. S4F, lanes 1–3 vs. 4–6). The EtOH challenge caused PRX1 levels to increase in the liver (Fig. S4F, lanes 1–6 vs. 7–12) and the degree of increase was greater with fortilin^Liver-Tg^ than fortilin^Liver-WT^ mice (Fig. S4F, lanes 7–9 vs. 10–12). It is thus likely that fortilin increased PRX1 activity in the EtOH-challenged liver by both mechanisms (a) and (b) (Fig. 4H).

## Discussion

We previously showed that fortilin binds p53 and protects cells against p53-mediated apoptosis and that the protective effects of fortilin against UV-irradiation-induced apoptosis are entirely dependent on p53^27^. In the current study, we found that fortilin protected both U2OS cells and p53-null SAOS cells from H_2_O_2_-induced apoptosis (Fig. 1A), suggesting that the protective effect of fortilin against H_2_O_2_ is mediated through a pathway other than that of p53. We used a large-scale, unbiased, immunoprecipitation-coimmunprecipitation system to identify protein partners of fortilin and to explore how fortilin protects cells against ROS-induced apoptosis. We found that fortilin specifically interacts with PRX1 (Fig. 1B–I), a member of the peroxiredoxin family that contains two essential catalytic cysteine residues and uses thioredoxin as an electron donor. PRX1, abundantly expressed in all cells^32^, plays a critical role in the scavenging of ROS; mice lacking PRX1 exhibit a shortened lifespan due to severe hemolytic anemia, due to the ROS-induced damage in the erythrocyte plasma membrane^56^.

*PRX1* is transcriptionally activated by Nrf2 in the context of hypoxia and reoxygenation^57^,^58^. The activity of PRX1 protein is negatively regulated by phosphorylation of its threonine residues by Mst1^28^ and CDKs such as Cdc2^45^. However, it had not been known how phosphorylation by the kinases is regulated or how the degradation of PRX1 is controlled. Our work presented here establishes that fortilin keeps PRX1 enzymatically active in two ways (Fig. 4H). First, fortilin protects PRX1 from ubiquitination (Figs S2D & S4G) and proteasome-mediated degradation (Fig. 2A). The protection by fortilin against ubiquitination and proteasome-mediated degradation is also shown with MCL1, a Bcl-2 family member pro-survival molecule^38^. Second, the virtual docking experiment (Fig. 3D–F & Fig.S3) suggested that fortilin covers the key phosphorylation sites of PRX1—Thr^90^ and Thr^183^—thereby preventing Mst1, a PRX1 kinase, from accessing, phosphorylating, and deactivating PRX1 (Fig. 3A,C).

In mice specifically overexpressing fortilin in the liver, fortilin collaborated with PRX1 to protect the liver against alcohol-induced injury (Fig. 4B–D). The overexpression of fortilin in the liver decreased the deactivating phosphorylation of PRX1 and enhanced its peroxidase activity (Fig. 4E,F&G). Tissue-specific overexpression of fortilin was critical for our ability to test this hypothesis. We previously attempted to generate transgenic mice constitutionally overexpressing fortilin by using the traditional approach of injecting a fortilin transgene into blastocysts for random integration of the transgene into the genomic DNA. This approach resulted in mice with fortilin protein expression barely above that of wild-type mice (data not shown). We therefore turned to the HPRT-targeting strategy described by us and others^48^,^59^ to insert a single-copy fortilin transgene into a locus that drives robust expression of the gene at a specific location. This approach was successful, and fortilin expression in the liver was by far greater in fortilin^Liver-Tg^ mice than in their wild-type counterparts (fortilin^Liver-WT^)(Fig. S4B).

Ethanol is metabolized in the liver through three major pathways—alcohol dehydrogenase in the cytosol, microsomal ethanol oxidizing system in the endoplasmic reticulum, and aldehyde oxidase in the mitochondria^60^. These three distinct pathways of metabolizing ethanol inevitably produce reactive oxygen species (ROS) such as superoxide, hydroxyl radical, and hydrogen peroxide^60^. Extracts from the liver, but not the brain, spleen, or kidney, of ethanol-fed animals, contained a large amount of ROS as determined by electron spin resonance (ESR) spectroscopy^61^. Hepatic superoxide anion production increased 7-fold and reached a peak at 3 hours after acute alcohol ingestion in rats when measured in the perfused rat liver^62^. Acute doses of alcohol, but not placebo, drastically and dose-dependently increased the level of urinary isoprostanes, which are free radical-catalyzed products of arachidonic acid in humans^63^. These reports support that acute alcohol ingestion causes excessive and rapid production of ROS and leads to oxidative damage in the liver. We thus used the acute alcohol-induced liver injury model to evaluate whether fortilin collaborates with PRX1 to protect the liver against ROS-mediated damage. Acute (short-term) high-dose, not chronic (long-term) low-dose, alcohol feeding was performed to (a) mitigate the possibility of alternation of alcohol metabolism by fortilin overexpression and (b) increase the chance of detecting the primary phenotype caused directly by fortilin overexpression, and not those caused indirectly by the perturbation of various genes from chronic and long-term administration of alcohol.

The protection of PRX1 by fortilin and the collaboration between fortilin and PRX1 to reduce alcohol-induced liver injury have several important clinical implications. Excessive alcohol consumption is the third leading preventable cause of death in the United States. Among various causes of alcohol-related death, alcoholic liver disease represents the most significant cause of mortality, to which 44% of all deaths from liver disease are attributed. Affecting predominantly younger people, approximately thirty (30) years of life are lost per alcohol-related death, which translates to about 2.3 million years of potential life lost per year^51^. Acute alcoholic hepatitis, simulated in the experiments described in Fig. 4, is the most catastrophic type of alcoholic liver disease, manifesting itself in sudden onset of severe liver impairment often following a short-term alcoholic binge and it is associated with a mortality rate of up to 60%^52^. Alcohol causes liver damage through the generation of ROS and subsequent peroxidation of lipids, DNA, and proteins as evidenced by the fact that mice lacking Cu, Zn-superoxide dismutase (SOD1) exhibit extensive oxidative liver damage in response to alcohol consumption^64^. Our results suggest that pharmacological strategies to increase the hepatocellular fortilin concentrations could protect the liver from alcohol-induced, ROS-mediated damage in humans. Such pharmacological strategies might include micro-RNAs and small molecules that increase fortilin levels in hepatocytes. In addition, such strategies could be effective in preventing other ROS-induced liver damage such as that seen in ischemia-reperfusion injury associated with liver transplant surgery^65^ and acetaminophen overdose^66^.

The current data set does not allow us to clearly determine how much of the protective activity of fortilin against alcohol-induced liver injury originates from its anti-oxidant function as opposed to its canonical anti-apoptotic function. In addition, we do not know whether fortilin also regulates other PRXs. Co-immunoprecipitation experiments showed that fortilin also interacts with PRX-2, PRX-3, and PRX-5 (Fig. S1E). It is possible that fortilin also protects these PRXs against proteasome-mediated degradation and Mst1-mediated phosphorylation and deactivation, although this needs to be experimentally evaluated. Further, although the liver lysate from fortilin^Liver-Tg^ mice showed more peroxidase activity than lysates from fortilin^Liver-WT^ mice (Fig. 4E), we do not know if this is solely due to enhancement of PRX1 activity, because all 6 PRXs are expressed in the liver (data not shown).

Finally, our current work shows that fortilin also functions as a redox molecule, exerting its activity through its synergistic binding to PRX1, an anti-ROS protein. The newly uncovered interaction between fortilin and PRX1 could be therapeutically exploited to protect cells against ROS-induced apoptosis not only in the liver but also in other organs. The fortilin^Tg/−^ mice described here (Fig. S4A) should be a valuable tool for evaluating the role of fortilin and the fortilin-PRX1 interaction in handling ROS in a tissue-specific fashion.

## Materials and Methods

### Molecular cloning

*Cloning of PRX1 into the CMV-FLAG-vector:* The human *PRX1* cDNA sequence (NCBI Accession No. AAH21683.1) was directionally cloned into the p3X-FLAG CMV14 vector (Sigma-Aldrich, St. Louis, MO) using the following PCR primers: Forward: 5′-GC*GAATTC*GCGATGTCTTCAGGAAATGCT-3′ and Reverse: 5′GCG*GGATCC*GCGCTTCTGCTTGGAGAAATATT-3′ (the *Eco*RI and *Bam*HI sites in the primers are italicized). Cloning of PRX2, PRX3, PRX4, PRX5, and PRX6 into the p3X-FLAG CMV14 vector was accomplished using the same methods. *Cloning of fortilin cDNA into the pESG-IBA5-vector:* The cDNA encoding human fortilin was cloned into the multiple cloning site of the pESG-IBA5 mammalian expression vector (IBA Life Sciences, Gottingen, Germany) by using a PCR-based strategy. *Cloning of fortilin cDNA into the pLV-CMV-MCS-PGK-Puro-vector:* The cDNA encoding human fortilin was cloned into the multiple cloning site (MCS) of the pLV-CMV-MCS-PGK-Puro- mammalian expression vector (CMV = cytomegalovirus promoter; PGK = 3-phosphoglycerate kinase promoter; Puro = puromycin resistant gene) (Cellomics Technology, Halethorpe, MD) using a PCR-based strategy.

### Cell culture and cell lines

The U2OS, SAOS and 293T cell lines were purchased from the American Type Culture Collection (ATCC, Manassas, VA). All cell lines were maintained in high-glucose Dulbecco’s modified Eagle’s medium (DMEM) and supplemented with 10% fetal bovine serum (FBS) at 37 °C in an atmosphere containing 5% CO_2_. U2OS_Lenti-fortilin_, U2OS_Lenti-empty_, SAOS_Lenti-fortilin_, and SAOS_Lenti-Empty_ were generated by cotransfection of lentiviral vectors (pLV-CMV-fortilin-PGK-Puro and pLV-CMV-empty-PGK-Puro) and packaging plasmids into 293T cells, followed by ultracentrifugation of viral supernatant as described previously^67^ (Cellomics Technology). The cell lines were maintained in DMEM supplemented with 10% FBS and Puromycin (2.5 μg/mL, Mediatech, Inc, Manassas, VA). U2OS_Fortilin-HA, PRX1-FLAG_ was generated by stably transfecting U2OS_Fortilin-HA_ with the pCMV14-FLAG-PRX1 plasmid vector (Sigma) and monoclonally selecting the cells that express both PRX1-FLAG and Fortilin-HA using Zeocin and G418.

### Western blot analyses

Western blot analyses were performed as we described previously^11^,^17^,^18^,^20^,^23^, using the following antibodies: anti-fortilin (polyclonal antibody, MBL International, Woburn, MA, used for Fig. 1A,B, Fig. 2A, Fig. 3C, Fig. S2B, Fig. S2C, Fig. S2D and Fig. S4B; monoclonal antibody [Clone 2C4], Abnova, Taiwan used for Fig. 1F, Fig. 3A and Fig. S4F), anti-hemagglutinin (HA; 16B12, Bethyl Laboratory, Montgomery, TX), anti-FLAG (M2, Sigma), anti-human p53 (DO1, Santa Cruz), anti-PRX1 (goat polyclonal, PAB11441, Abnova, Taiwan), anti-glyceraldehyde-3-phosphate dehydrogenase (GAPDH; 6C5, Fitzgerald), anti-ubiquitin (BostonBiochem, Cambridge, MA), and anti-phosphothreonine (Millipore, Billerica, MA) antibodies.

### Real-time quantitative reverse transcription polymerase chain reaction (RT-qPCR)

We have previously described the methods of RT-qPCR^27^. Briefly, the organs or cells were harvested into Tri-Reagent (Molecular Research Center, Cincinnati, OH). RNA was isolated in accordance with the manufacturer’s instructions and treated with DNAse (ABI, Foster City, CA). RT-qPCR was performed in quadruplicate with exactly 50 ng of total RNA, using the TaqMan® RT-PCR kit (Applied Biosystems [ABI] at Life Technologies, Grant Island, NY) in the ABI Step One Plus Real-Time PCR system and the following primer and probe sets (Integrated DNA Technologies, Coralville, IA):

1. Mouse PRX1—Forward: 5′- ACAAGGAGGATTGGGACCCATGAA -3′, Reverse: 5′-TAATCTCATCCACAGAGCGGCCAA-3′, Probe: 5′-FAM- AGCGCACCATTGCTCAGGATT-IABkFQ-3′ where FAM = carboxyfluorescein and IABkFQ = Iowa Black FQ
2. Mouse fortilin— Forward: 5′-TCCGACATCTACAAGATCCGG-3′, Reverse: 5′- ATCTTGCCCTCCACCTCCA-3′, Probe: 5′-FAM-AGATCGCGGACGGGCTGTGC-IABkFQ-3′
3. Mouse GAPDH— Forward: 5′-TGTGATGGGTGTGAACCACGAGAA-3′, Reverse: 5′-GAGCCCTTCCACAATGCCAAAGTT-3′, Probe: 5′-JOEN-ATTGCATCCTGCACCACCACCTGCTT-IABRQSP-3′ where JOEN = 6-carboxy-4;,5′-dichloro-2′,7;-dimethoxyfluorescein and IABRQSP = Iowa Black Rq-Sp.
4. Human PRX1— Forward: 5′-CGGGCCTCTAGATCACTTCT-3′, Reverse: 5′-TATGTCTTCAGGAAATGCTA-3′, Probe: 5′-FAM-AGCGCACCATTGCTCAGGATT-IABkFQ-3′.
5. Human GAPDH— Forward: 5′-GCGAGATCCCTCCAAAATCAA-3′, Reverse: 5′-GTTCACACCCATGACGAACAT-3′, Probe: 5′-JOEN- CAAGCTTCCCGTTCTCAGCC-IABRQSP-3′

### DNA fragmentation assay

The Cell Death Detection ELISA PLUS kit (Roche, Indianapolis, IN, Catalog #: 11774425001) was used in accordance with the manufacturer’s instructions, with modifications described previously^27^. Cells (5 × 10^5^) were seeded into each well of a 6-well plate. The next morning, cells were treated with phosphate-buffered saline (PBS) or 500 μM H_2_O_2_ in PBS for 4 hours before they were harvested (both adherent and floating) and subjected to the DNA fragmentation assay^27^.

### Large-scale immunoprecipitation to identify protein partners of fortilin

Cleared lysate containing 10 mg of protein from U2OS_Empty-HA_ or U2OS_Fortilin-HA_ cells was obtained after lysing the cells in lysis buffer (20 mM HEPES [pH = 7.4], 35 mM NaCl and 0.001% NP-40). The lysates were mixed with anti-HA agarose beads and incubated overnight at 4 °C on an end-over-end rotator, followed by four washes with wash buffer (20 mM HEPES [pH = 7.4], 150 mM NaCl and 0.01% NP-40) for 10 minutes each. The mixtures were eluted into 4x SDS loading buffer and boiled for 5 minutes. The proteins were resolved on a large-format 10% SDS polyacrylamide gel and stained with SYPRO Ruby. The protein bands were visualized under UV light. Protein bands observed differentially in the U2OS_Fortilin-HA_ and U2OS_Empty-HA_ lanes were immediately excised and submitted for identification by Matrix Assisted Laser Desorption Time-of-Flight (MALDI-TOF/TOF) mass spectrometry at the UTMB Biomolecular Resource Facility.

### Immunoprecipitation and co-immunoprecipitation

We previously described the details of the immunoprecipitation and co-immunoprecipitation procedures^27^. For cell-based forward immunoprecipitation (Fig. 1D), cleared total cell lysates from U2OS_Empty-HA_ and U2OS_Fortilin-HA_ were incubated with agarose-conjugated anti-HA (clone 3F10, Roche). Formed complexes were precipitated by centrifugation, washed four times, eluted into SDS gel loading buffer, and subjected to SDS-PAGE, Western blot transfer, and immunodetection using anti-HA (16B12; Bethyl Laboratories, Montgomery, TX) and anti-PRX1 (polyclonal goat, Abnova, Taiwan) antibodies. For cell-based bi-directional immunoprecipitation (Fig. 1E and Fig. S1E), cleared lysates from U2OS_Fortilin-HA, PRX1-FLAG_ (for Fig. 1E; U2OS_Fortilin-HA, PRX2-FLAG_, U2OS_Fortilin-HA, PRX3-FLAG_ U2OS_Fortilin-HA, PRX4-FLAG_ U2OS_Fortilin-HA, PRX5-FLAG_, or U2OS_Fortilin-HA, PRX6-FLAG_ for Fig. S1E) were divided into three microfuge tubes each, which contained bare agarose beads, agarose beads conjugated with an anti-FLAG (M2) antibody, or agarose beads conjugated with an anti-HA antibody. The reaction mixtures were incubated at 37 °C for 4 hours before the formed complexes were washed, eluted into SDS loading buffer, and subjected to Western blot analysis using anti-HA and anti-FLAG antibodies. For native immunoprecipitation (Fig. 1F), the cleared total lysate from the C57BL/6J mouse liver was divided equally into two microfuge tubes to which 10 μg of mouse anti-PRX1 antibody (13E7, Abcam, Cambridge, MA) or 10 μg of normal mouse IgG was added, followed by sheep anti-mouse magnetic Dynabeads® (Novex by Life Technologies, Oslo, Norway). The tubes were allowed to incubate overnight at 4 °C on an end-over-end rotating platform. The beads were then collected by brief centrifugation and application of a magnetic field and then washed three times with lysis buffer (20 mM HEPES [pH = 7.4], 35 mM NaCl and 0.001% NP-40) for 5 minutes each. The immunoprecipitated protein complexes were then eluted by treatment with 4x SDS loading buffer for 20 minutes at room temperature, following which the eluate was collected by application of a magnetic field to the beads. The eluate was then boiled for 5 minutes and subjected to immunodetection using anti-fortilin and anti-PRX1 antibodies. For evaluation of the phosphorylation status of liver PRX1 (Fig. 4G), we first conjugated anti-PRX1 antibody (Clone 2A4, Pierce Antibodies, Waltham, MA) to Protein A/G agarose beads using the AminoLink® Plus Immobilization Kit (Pierce), according to the manufacturer’s instructions. Next, liver tissue from fortilin^Liver-WT^ and fortilin^Liver-Tg^ mice was lysed in Lysis Buffer (20 mM HEPES, pH = 7.4, 35 mM NaCl, and 0.001% NP-40) supplemented with Complete Protease Inhibitors (Roche, Indianapolis, IN) and phosphatase inhibitors (Sigma-Aldrich, St. Louis, MO) and cleared by centrifugation (16,000 g for 15 min at 4 °C). Immunoprecipitation of PRX1 was achieved by incubating 10 mg of proteins from each animal with the above anti-PRX1-Protein A/G agarose beads overnight at 4 °C. On the next day, beads were collected by centrifugation, and washed three times with Lysis Buffer. Proteins bound to the beads were eluted in 4xSDS loading buffer at 95 °C for 5 min and resolved by SDS-PAGE before being subjected to Western blot analyses using anti-PRX1 (Abnova) and anti-phosphothreonine (Millipore) antibodies. The degree of threonine-phosphorylation of PRX1 was assessed by the PRX1 phosphorylation index, which was calculated by dividing the signal intensity of the phosphothreonine band by that of the respective total PRX1 band and was expressed as arbitrary units (A.U.). Statistical analysis was performed based on three independent experiments to test the hypothesis that the phosphorylation status of PRX1 at its threonines was lower in fortilin^Liver-WT^ liver than in fortilin^Liver-Tg^ liver.

### Immunocytochemistry of fortilin and PRX1

Immunocytochemical analyses were performed as we described previously^20^. In brief, U2OS cells were seeded on a cover glass, fixed in 10% buffered formalin solution for 5 min, permeabilized in 0.1% Triton X, and incubated with mouse anti-fortilin (Clone 2C4, Abnova, Taiwan) and goat polyclonal anti-PRX1 (PAB11441, Abnova, Taiwan) antibodies. After washes, bound antibodies were detected with donkey anti-mouse AlexaFluor® 488-conjugated and donkey anti-goat AlexaFluor® 568-conjugated (Invitrogen, Grand Island, NY) secondary antibodies, respectively. DAPI was used to counterstain the nuclei. The stained slides were examined under a confocal microscope (LSM 510 Meta, Zeiss, Germany) with appropriate filter sets.

### Proximity ligation assay

The method was originally described by Soderberg *et al.*^37^ Wild-type U2OS cells seeded on a chamber slide were fixed in 10% buffered formalin solution, permeabilized in 0.1% Triton X, and incubated with primary mouse anti-fortilin (2C4, Abnova) and goat anti-PRX1 (Abnova) antibodies. The chamber slide was then incubated for 1 hour with secondary anti-mouse and anti-goat antibodies conjugated to oligonucleotides (PLA probes MINUS and PLUS, Duolink *In Situ* Proximity Ligation Assay, Sigma-Aldrich) before ligase and two connector oligonucleotides were added to the solution. These oligonucleotides would hybridize to the two PLA probes and join them into a closed circle if they are in close proximity (30 nm). Subsequently, fluorescently labeled oligonucleotides that hybridize to the rolling circle amplification product were added. A Zeiss LSM 510 Meta confocal microscope system (Zeiss, Germany) was used to visualize the signals.

### Generation of recombinant human fortilin

Affinity purification of human recombinant fortilin was performed by using the Strep-tag purification system (IBA Life Sciences, Goettingen, Germany)^39^. We performed trypsinization and centrifugation to collect 1 × 10^9^ 293T cells stably expressing human fortilin tagged with the Strep-tag II (WSHPQFEK) at its N-terminal end, washed them in PBS, resuspended them in Buffer W (100 mM Tris HCl [pH = 8], 150 mM NaCl, 1 mM EDTA), lysed them by repeated freeze-thaw cycles, and sonicated them to shear the genomic DNA. Cleared total cell lysate was then passed through a column packed with Strep-Tactin-Superflow resin. The column was washed five times with Buffer W before the Strep-tagged fortilin was eluted with Buffer E (Buffer W plus 2.5 mM desthiobiotin). Recombinant human fortilin was then characterized by Coomassie and Western blot analyses (Fig. S2B). Finally, the fractions were pooled and concentrated using centrifugal filters (Amicon® EMD Millipore, Billerica, MA). The concentrated protein samples were buffer-exchanged into PBS by using Zeba™ Spin Desalting Columns (Thermo Scientific, Waltham, MA).

### Biolayer interferometry

Recombinant fortilin protein produced as described above was biotinylated and immobilized on streptavidin-coated biosensors (ForteBio, Menlo Park, CA) at a concentration of 1 μg/mL in BI Buffer (25 mM Tris, 150 mM NaCl, 0.1% Tween-20) for 600 seconds, followed by buffer exchange into PBS. We then added various concentrations of recombinant PRX1 (Sigma-Aldrich, 0 to 5000 μM) for 180 seconds to evaluate the association between the two molecules. Finally, we replaced the solution with PBS for 300 seconds to evaluate their dissociation. The binding data were processed and a dissociation constant was calculated by using BLItz analysis software (Forte Bio).

### PRX1 degradation assay

U2OS_sh-Control_ and U2OS_sh-Fortilin_ cells (2 × 10^5^ of each) were plated in each well of a 6-well plate and allowed to incubate overnight at 37 °C. The next morning, the cells were washed with PBS; exposed to culture medium containing 100 μg/mL cyclohexamide (CHX) with or without 20 μM of MG132, a proteasome inhibitor; and harvested into RIPA buffer at 0, 2, 4, 8, 12 and 24 hours after CHX treatment. The culture medium was replaced every 12 hours to replenish CHX, which degrades after 12 hours. The lysates (10 μg each) were subjected to Western blot analysis using anti-PRX1 and anti-GAPDH antibodies. Band intensities of the proteins were quantified using the LI-COR imaging system software (LI-COR Biotechnology, Lincoln, Nebraska). The PRX1 expression index was calculated as the ratio of signal intensities of the PRX1 and GAPDH bands at each data point.

### PRX1 activity assay

We used the method described by Kim *et al.*^40^ to determine the peroxidase activity of PRX1 *in vitro*. For PRX1 to exert its peroxidase activity (i.e., the reduction of an H_2_O_2_ molecule to H_2_O molecules), PRX1 needs to be kept in its reduced form. This is achieved by the reduction of oxidized PRX1 by thioredoxin (Trx) to its reduced state. The now-oxidized Trx then needs to be reduced by thioredoxin reductase (TrxR) using a NADPH molecule before it can again regenerate reduced PRX1 from oxidized PRX1. Thus, the reduction in NADPH concentration in the reaction mixture—as monitored by absorbance at 340 nm—correlates with PRX1′s peroxidase activity (Fig. 2C, the top panel). In the actual assay, we generated a reaction mixture in 50 mM HEPES-NaOH buffer (pH 7.0) by adding 200 μM NADPH, 3 μM recombinant Trx (Sigma-Aldrich, St. Louis, MO), and 1.5 μM TrxR. Either wild-type fortilin or fortilin_ΔL7R_, along with PRX1 when appropriate, was added to the reaction mixture. The reaction was initiated by adding 100 μM H_2_O_2_, maintained at 30 °C, and monitored for 30 min by following the reduction in absorbance at 340 nm in a SpectraMax M5 spectrophotometer (Molecular Devices, Sunnyvale, CA). In addition to plotting absorbance at 340 nm against elapsed time, we calculated the initial rate of the reaction from the linear portion of the above curve and expressed it as the amount of NADPH oxidized per minute. More specifically, the data points of the first 8 min of each reaction mixture were subjected to regression analysis to generate a linear regression line. The initial rate of the reaction was expressed in arbitrary units (A.U.) as (the slope of the regression line)*(-1)*1000.

### PRX1 phosphorylation assay

Recombinant human Mst1 (400 ng; ProQinase GmBH, Freiburg, Germany) and PRX1 (2 μg; Sigma-Aldrich, St. Louis, MO) were incubated in kinase buffer (50 mM Tris-HCl [pH 7.4], 10 mM MgCl_2_, supplemented with 1 mM dithiothreitol and 20 μM ATP immediately before use) in the presence of increasing amounts of fortilin or fortilin_ΔL7R_ (0, 1, 2, and 4 μg) at 30 °C for 30 min before the reaction mixture was subjected to Western blot analysis using an anti-phosphothreonine antibody (Cell Signaling Technology, Beverly, MA).

### Docking study

DockoMatic V 2.0 is a graphical user interface that facilitates the use of AutoDock V 4.2 as a docking engine to identify energetically favorable molecular interactions. For the purposes of the present investigation, rigid docking was performed between fortilin and PRX1. The use of rigid docking provided qualitative output that was not intended to be interpreted as a quantitative measure of thermodynamic parameters to describe the interaction between these proteins. In this study, we predicted favorable orientations for molecular assemblies involving fortilin and PRX1. First, the crystal structures for fortilin (PDB ID: 2HR9) and PRX1 (2RII) were obtained from the Research Collaboratory for Structural Bioinformatics (RCSB)^68^. A grid parameter box was created that encompassed Leu^7^ and the Leu^7^ side of the fortilin protein. A screening run consisting of 100 simulations identified two principal clusters for PRX1 docking conformations to fortilin on the Leu^7^ side of the protein. A second, more exhaustive exploration of the system was conducted by increasing the number of simulations to 500. This latter experiment returned a result consistent with that from the screening run. Two principal clusters were observed to represent the docking of PRX1 to fortilin; the more energetically favored of the two clusters was reinforced by this more exhaustive sampling. There were 120 PRX1 molecules in a similar orientation in the less favored cluster and 159 bound PRX1 molecules in the more favorable orientation in the second cluster. This latter fortilin-PRX1 complex served as the template upon which a second PRX1 monomer was introduced to form the PRX1 dimer. Because the crystal structure of the monomer used for docking was a truncated version of the dimer, the second PRX1 monomer could be reintroduced in the proper orientation as detailed in the RCSB protein database file. Due to the rigid docking experiments, it was deemed necessary to provide flexible binding for the C-terminal tail of the PRX1 molecule. The tail, which consists of the last 16 amino acids at the C-terminus of PRX1, was allowed to bind across the entire surface of the fortilin molecule, independent of any other molecular interactions. Of 500 simulations, a cluster consisting of 193 similar orientations emerged as the most favorable docking pose for the tail. The docked tail was then used as an anchor point for attachment to the rest of the PRX1 protein for which the most favorable docked pose was conserved. The last step was to include the second PRX1 molecule to provide the PRX1 dimer bound to fortilin.

### Generation of mice with a liver-specific fortilin transgene

To generate liver-specific fortilin transgenic mice, an inducible stop-lox approach was used as described by Soriano^47^. We first constructed a fortilin conditional transgene cassette (fortilin^Tg^) consisting of (a) CAG promoter, (b) Kozak-ATG sequence, (c) the first LoxP sequence, (d) neomycin resistance gene with a stop codon, (e) the second LoxP sequence, (f) mouse fortilin complementary DNA (cDNA), (g) stop codon, and (h) the poly-A sequence. The CAG promoter, consisting of the cytomegalovirus intermediate early enhancer and a modified chicken β-actin promoter, was originally described by Niwa *et al.*^69^ and represents a strong synthetic mammalian gene expression promoter.

We then cloned the fortilin^Tg^ cassette into the pENTR™ 1A plasmid (Life Technologies) by using a PCR-based strategy. We then inserted the fortilin^Tg^ cassette from the pENTR™ 1A plasmid into a Gateway® destination vector (pDEST) containing the homology arms for the hypoxanthine phosphoribosyltransferase (HPRT) locus by using the LR-Clonase®-mediated *in vitro* recombination strategy in accordance with the manufacturer’s instructions (Life Technologies).

The fortilin^Tg^ cassette on the targeting vector was extensively sequenced to verify the lack of mutation. The vector was then linearized and electroporated into HPRT-deficient male BPES cells (coat-color = agouti). Successful integration of the fortilin transgene through homologous recombination corrected the HPRT deficiency and allowed the BPES cells to grow in the hypoxanthine aminopterin-thymidine-supplemented (HAT) selective medium. Three positive BPES cell clones were selected by a PCR-based strategy, verified by Southern blot analyses for a single integration, and were microinjected to C57BL/6J blastocysts (coat-color = black) to generate chimeras. These blastocysts were then transplanted into CD1 female mice rendered pseudopregnant by mating to a vasectomized male. The resulting chimeras were identified first by the presence of agouti pigmentation in their fur because agouti pigmentation represents contributions from the ES cells. Male chimeras were subsequently mated to C57BL/6J females to generate a line of transgenic mice. The resultant agouti pups were genotyped with PCR and pups with germline transmission were identified.

Because the HPRT locus is on the X chromosome, transgenic males are hemizygous containing one copy of the transgene while transgenic females could have one or two transgenic alleles. In the current work, we exclusively used male mice. These transgene knock-in mice were referred to as fortilin^Tg/−^ or fortilin^WT/−^ in males (the superscripted “-“ here denotes the lack of the 2^nd^ X chromosome in males).

Over-expression of fortilin in the liver was triggered *in vivo* by crossing these animals with C57BL/6J mice overexpressing the Cre-transgene under the control of the liver-specific albumin promoter (Alb-Cre^+/+^ mice, The Jackson Laboratories). We found that fortilin transgene expression was significantly higher in Alb-Cre^+/+^ fortilin^Tg/−^ mice than in Alb-Cre^+/−^fortilin^Tg/−^ mice, suggesting that high expression of Cre-recombinase is required for removal of the LoxP-neo-stop-LoxP sequence. We therefore performed all subsequent experiments using male Alb-Cre^+/+^ fortilin^Tg/−^ (denoted fortilin^Liver-Tg^ hereafter) and Alb-Cre^+/+^ fortilin^WT/−^ (denoted fortilin^Liver-WT^) mice.

### Mouse model of alcohol-induced, reactive-oxygen-species-mediated, liver injury

Alcohol was diluted in PBS at 20% v/v. We induced reactive-oxygen-species-mediated tissue injury in the livers of C57BL/6J male mice (12 weeks of age) by administering 10 g/kg of alcohol divided into 12 doses that were given every 3 hours via oral gavage. Thirty-six hours after administration of the last alcohol dose, the mice were sacrificed by carbon dioxide intoxication and cervical dislocation, blood was collected by cardiocentesis, and organs were harvested for further analyses. Blood was subjected to alanine aminotransferase (ALT) determination and the liver tissue was stained with terminal deoxynucleotidyl transferase dUTP nick end labeling (TUNEL)^70^, α-4-hydroxynonenal (4-HNE), and fragmented cytokeratin-18 (fCK-18). The liver was also assayed for tissue malondialdehyde (MDA) level and Prx peroxidase activity^40^.

### TUNEL staining

TUNEL staining was performed as previously described^16^,^27^ by using the FragEL^TM^ DNA Fragmentation Detection Kit (Calbiochem) in accordance with the manufacturer’s instructions. At least 600 cells were counted and TUNEL indices were calculated as the number of TUNEL-positive cells divided by the number of total cells counted, and expressed as percentages.

### Serum alanine transaminase (ALT) assay

Serum ALT was quantified as we previously described^16^.

### MDA assay

Tissue MDA levels were determined as described previously^53^.

### Immunohistochemistry of mouse liver

Immunohistochemistry of mouse liver was performed as we described previously^70^ using antibodies against 4-HNE (HNEJ-2, Abcam, Cambridge, MA), and fCK-18 (Clone M30, Peviva, Nacka, Sweden), with 3,3′-diaminobenzidine (DAB) as the chromogen. fCK-18 indices were determined as the number of fCK-18-positive cells divided by the total number of cells counted, and expressed as percentages. 4-HNE indices were calculated as the DAB-positive area divided by the region of interest and expressed as percentages, as we previously described^70^. In addition to expressing the degree of staining in continuous values as defined above in the dot plots, we employed a scoring system to display the same data in a more intuitive visual fashion. More specifically, Score 0 represented the samples that are in the first quartile, Score 1 the second quartile, Score 2 the third quartile, and Score 3 the fourth quartile.

### Two-dimensional (2D) gel analysis of mouse liver proteins

Proteins were extracted from mouse livers by using lysis buffer (20 mM HEPES [pH 7.5], 35 mM NaCl and 0.001% NP-40, supplemented with complete protease inhibitor cocktail and phosphatase inhibitor cocktail), followed by sonication four times using 15-second pulses. A Bradford assay (Bio-Rad, Hercules, CA) was used to estimate protein concentration. For each sample, 125 μg of total protein was acetone-precipitated at −80 °C overnight to remove the extraction buffer. The protein pellet was resuspended in 2D urea buffer and separated by using 2D gel electrophoresis as we previously described^71^. The 2D/Western blot analysis was performed to separate liver proteins first based on their isoelectric points (pI) using an Ettan IPGphor Isoelectric Focusing System (GE Healthcare, Pittsburg, PA), and then based on their molecular weight in 10% SDS-PAGE gels (Bio-Rad).

### Alkaline phosphatase treatment

Liver lysates (125 μg protein/sample) were acetone-precipitated and the protein pellet was resuspended in calf intestinal phosphatase (CIAP) buffer (100 mM NaCl, 50 mM HEPES [pH 7.5], 10 mM MgCl_2_, 1 mM dithiothreitol, complete protease inhibitor cocktail and 0.4% NP-40). Samples were then either treated with buffer only or treated with CIAP (New England BioLabs, Ipswich, MA; 1 unit of CIAP per 1 μg of protein) at room temperature for 30 minutes. The CIAP- and buffer-treated samples were subjected to 2D gel electrophoresis as described above, followed by Western blotting using a PRX1-specific antibody (goat polyclonal, Abnova). The loading and transfer conditions of each nitrocellulose membrane were evaluated with Ponceau S staining (Sigma).

### Ubiquitination assay

***Cellular ubiquitination assay***was performed as follows. 0.25 × 10^6^ each of U2OS_Lenti-fortilin_ and U2OS_Lenti-empty_ cells were seeded onto 2 wells each of a 6-well plate, transfected with either (a) pcDNA3-HA-Ubiquitin and pCMV14-FLAG-PRX1-or (b) pcDNA3-HA-Ubiquitin only (1 μg each), using X-tremeGENE9 (Roche Life Science, Indianapolis, IN). Twenty-four hours after transfection, the cells were washed with PBS, harvested directly into 100 μL of 4XSDS Loading Buffer, sonicated, and then boiled at 95 °C for 5 min. 10 μL of the lysates were subjected to SDS-PAGE and Western blot analysis as described above, using anti-HA, anti-FLAG, anti-GAPDH, and anti-fortilin antibodies. The degree of PRX1 ubiquitination was assessed by the PRX1 ubiquitination index, calculated by dividing the signal intensity of the higher-molecular-weight bands of the anti-FLAG blot (indicated in the figure) by that of respective PRX1 band and expressed as arbitrary unit (A.U.). ***In vivo ubiquitination assay*** was performed as follows. Liver tissue from fortilin^Liver-WT^ and fortilin^Liver-Tg^ mice was lysed in Lysis Buffer (20 mM HEPES, pH = 7.4, 35 mM NaCl, and 0.001% NP-40) supplemented with Complete Protease Inhibitors (Roche, Indianapolis, IN), phosphatase inhibitors (Sigma-Aldrich, St. Louis, MO), and deubiqutinase inhibitors (U-201, U202, and U-203; Boston Biochem, Cambridge, MA). The crude lysates were then cleared by centrifugation (16,000 g for 15 min at 4 °C). Immunoprecipitation of PRX1 was achieved by incubating 10 mg of proteins from each animal with anti-PRX1-Protein A/G agarose beads (described above) overnight at 4 °C. Next day, beads were collected by centrifugation, and washed three times with Lysis Buffer. Proteins bound to the beads were eluted in 4xSDS loading buffer at 95 °C for 5 min and resolved by SDS-PAGE before subjected to Western blot analyses using anti-PRX1 (Abnova) and anti-ubiquitin (Boston Biochem, Cambridge, MA) antibodies. The degree of PRX1 ubiquitination was assessed by the PRX1 ubiquitination index, calculated by dividing the signal intensity of the ubiquitin bands by that of respective total PRX1 band and expressed as arbitrary unit (A.U.).

### Statistical Analysis

The degree of the spread of data was expressed by the standard deviation (±SD). Student’s *t*-test was used to compare the means of two groups. *P* < 0.05 was considered to be statistically significant.

### Study Approval

All experiments involving animals were approved by the Institutional Animal Care and Use Committee (IACUC) of the University of Texas Medical Branch (UTMB) and carried out in accordance with the approved guidelines.

## Additional Information

**How to cite this article**: Chattopadhyay, A. *et al.* Fortilin potentiates the peroxidase activity of Peroxiredoxin-1 and protects against alcohol-induced liver damage in mice. *Sci. Rep.* **6**, 18701; doi: 10.1038/srep18701 (2016).

## Supplementary Material

Supplementary Information
