# Supplementary material for: Fortilin potentiates the peroxidase activity of Peroxiredoxin-1 and protects against alcohol-induced liver damage in mice
Source: Sci Rep. 2016 Jan 4;6:18701. doi: 10.1038/srep18701 (PMC4698670; doi:10.1038/srep18701)
Supplement: Supplementary Information [file srep18701-s1.pdf]

Fortilin potentiates the peroxidase activity of Peroxiredoxin-1 and protects against alcohol-induced liver damage in mice

SUPPLEMENTARY INFORMATION

(REVISED MANUSCRIPT: Original Research to Scientific Reports)

Abhijnan Chattopadhyay, Decha Pinkaew, Hung Q. Doan, Reed B. Jacob, Sunil K. Verma, Hana Friedman, Alan C. Peterson, Muge N. Kuyumcu-Martinez, Owen M. McDougal, and Ken Fujise *

Contact Information: Ken Fujise, M.D., Division of Cardiology, University of Texas Medical Branch, 301 University Blvd, Suite JSA5.106G, Galveston, Texas, 77555. Telephone: 409-772-4885, Fax: 409-419-1777, E-mail Ken.Fujise@utmb.edu.

Supplemental Figure Legends

Figure S1 (Related to Figure 1). A. Protein sequence alignment of PRX1-6. The amino acid sequences of human PRX1-6 proteins were aligned to each other using the CLC Main Workbench (CLC Bio, Boston, MA).The level of conservation at each position in the alignment is shown in “Conservation”. The high of the bar reflects how conserved that particular position is in the alignment. If one position is 100% conserved, then the bar will be shown in full height. A consensus sequence for the alignment is shown in “Sequence logo” where for each amino acid residue, the largest letter represents the consensus residue and its height correlates with the degree of homology among the aligned sequences. Smaller letters may appear along with the consensus residue and represent less conserved but potentially significant homologies. Amino acids are colored by the RasMol amino color scheme according to the traditional amino acid properties. **B. PRX1 expression is ubiquitous and especially abundant in the kidney, liver, and lung**. Abbreviations: A.U., arbitrary unit. PRX1 mRNA levels were quantified by real-time quantitative reverse transcription polymerase chain reaction (RT-qPCR). **C. The anti-PRX1 antibody used in the current study is specific for PRX1.** I.B., immunoblot; α-PRX1, anti-PRX1 antibody; α-PRX2, anti-PRX2 antibody; α-PRX3, anti-PRX3 antibody; α-PRX4, anti-PRX4 antibody; α-PRX5, anti-PRX5 antibody; α-PRX6, anti-PRX6 antibody; N.A., not applicable; ±, minimum cross reactivity; -, no cross reactivity. Human recombinant PRX1 and proteins from U2OS cell total cell lysates were resolved in a 12% SDS gel, transferred to nitrocellulose membranes, and probed by the antibodies indicated in the figure. Anti-PRX1 antibody showed minimum cross

reactivity with other PRX proteins. **D. U2OS cells express both PRX1 and PRX4.** α-GAPDH, anti-glyceraldehyde 3-phosphate dehydrogenase antibody used as a loading control. **E. Fortilin physically interacts with PRX1, -2, -3, and -5.** IP, immunoprecipitation; HA, human influenza hemagglutinin tag; FLAG, DYKDDDDK-octapeptide tag; α-HA, anti-HA antibody; α-FLAG, anti-FLAG antibody; +, interaction present; -, interaction absent. Bi-directional in vivo co-immunoprecipitation experiments were carried out using U2OS cells overexpressing (a) HA-tagged fortilin and (b) FLAG-tagged PRX1-6. **F. Coomassie blue staining of human recombinant fortilin and PRX1 that were used for biolayer interferometry.** MWM, molecular weight marker.

Figure S2 (Related to Figure 2). A. Down-regulation of fortilin does not change PRX1 mRNA levels. Abbreviations: GAPDH**,** glyceraldehyde 3-phosphate dehydrogenase; $U2OS_{sh\text{-}Control}$, U2OS cells stably transfected with an empty shRNA lentiviral vector; $U2OS_{sh\text{-}Fortilin}$, U2OS cells stably transfected with an anti-fortilin shRNA lentiviral vector. **B. Purification and characterization of wild-type and mutant fortilins using an affinity chromatography system.** IB, immunoblot; α-Fortilin, anti-fortilin antibody; Fortilin-strep-tag, Strep-tagged fortilin protein; $Fortilin_{\Delta L7R}$-strep-tag, Strep-tagged mutant fortilin lacking PRX1 binding. Cleared total cell lysates from 293T cells overexpressing Fortilin-strep-tag or $Fortilin_{\Delta L7R}$-strep-tag were applied to a Strep-Tactin Sepharose column. After extensive washing, the Strep-tagged proteins were eluted with desthiobiotin and subjected to dialysis against phosphate-buffered saline (PBS). **C: The overexpression of fortilin does not change PRX1 protein levels in**

U2OS cells. U2OS cells were transduced by either control lentivirus expression vector or fortilin lentivirus expression vector, lysed in SDS-loading buffer, and subjected to SDS-PAGE and Western blot analysis in triplicates (N = 3 each). The loading condition was evaluated by GAPDH. U2OS cells transduced by fortilin lentivirus expression vector expressed significantly more fortilin than did U2OS cells transduced by control vector. The degree of PRX1 expression was calculated by dividing the signal intensities of PRX1 bands by those of GAPDH and expressed as the relative PRX1 expression index (PRX1/GAPDH [Arbitrary unit, A.U.]). There was no significant difference in the PRX1 expression index between fortilin-overexpressing and control U2OS cells (P = 0.10). **D: Fortilin decreases PRX1 ubiquitination in U2OS cells.** $U2OS_{Lenti\text{-}empty}$, U2OS cells harboring the control lentiviral vector; $U2OS_{Lenti\text{-}fortilin}$, U2OS cells overexpressing fortilin; HA-Ubiquitin, pcDNA3-HA-Ubiquitin vector; PRX1-FLAG, pCMV14-PRX1-FLAG vector; α-Fortilin, anti-fortilin antibody; α-GAPDH, anti-GAPDH antibody; α-FLAG, anti-FLAG antibody; α-HA, anti-HA antibody; Long Exp., long exposure to visualize poly-ubiquitinated PRX1s; Short Exp., short exposure to best visualized PRX1 without ubiquitination; PolyUb-PRX1, poly-ubiquitinated PRX1; Ub Index, PRX1 ubiquitination index. Lentiviral overexpression of fortilin decreased the poly-ubiquitinated PRX1s in U2OS cells as assessed by the PRX1 ubiquitination indices (Control vs. Fortilin = 1.0 vs. 0.36). See Materials and Methods for detail.

Figure S3 (Related to Figure 3). The PRX1 dimer interacts with a fortilin molecule. Abbreviations: Leu^7, the leucine residue in the seventh position in the fortilin

polypeptide; Thr^{90}, the threonine residue in the 90th position in the first PRX1 of the PRX1 dimer; Thr^{183}, the threonine residue in the 183rd position of the second PRX1 of the PRX1 dimer.

Figure S4 (Related to Figure 4). A. Generation of liver-specific fortilin transgenic mice. Abbreviations: HPRT, hypoxanthine-guanine phosphoribosyltransferase; ES cells, embryonic stem cells; LoxP, locus of X-over P1; CAG promoter, CMV early enhancer chicken beta actin promoter. **B. Characterization of mice overexpressing fortilinin the liver (fortilin$^{Liver\text{-}Tg}$ mice).** GAPDH, glyceraldehyde 3-phosphate dehydrogenase; RT-qPCR, real-time quantitative reverse transcription polymerase chain reaction; IB, immunoblot; α-fortilin, anti-fortilin antibody; α-GAPDH, anti-GAPDH antibody. Fortilin$^{Liver\text{-}Tg}$ mice (Alb-Cre$^{+/+}$fortilin$^{Tg/-}$), but not Alb-Cre$^{+/-}$fortilin$^{Tg/-}$ or Alb-Cre$^{-/-}$fortilin$^{Tg/-}$ mice, show drastically higher levels of fortilin mRNA and protein in the liver. **C. Fortilin$^{Liver\text{-}WT}$ and fortilin$^{Liver\text{-}Tg}$ mice have similarly low baseline levels of alanine transaminase (ALT).** NS, not statistically significantly different. **D. Fortilin$^{Liver\text{-}WT}$ and fortilin$^{Liver\text{-}Tg}$ mice have similarly low baseline levels of malondialdehyde (MDA).** A.U., arbitrary unit that is identical to what was used in **Fig.4C**. **E. Fortilin$^{Liver\text{-}WT}$ and fortilin$^{Liver\text{-}Tg}$ mice, without EtOH challenge, exhibit similar degrees of apoptosis and oxidative damage in the liver.** TUNEL, terminal deoxynucleotidyl transferase dUTP nick end labeling; fCK18, fragmented cytokeratin-18; 4HNE, 4-hydroxy nonenal; size bar = 100 μm; F.C., fold-change; NS, not statistically significant. Semiquantitative scoring, representative histochemistry, and quantitative measurements are shown. **F.**

Expression of PRX1 of the livers of fortilin$^{Liver\text{-}WT}$ and fortilin$^{Liver\text{-}Tg}$ mice treated by either PBS or ethanol. PBS, phosphate-buffered saline; EtOH, ethanol; IB, immunoblot; α-GAPDH, anti-GAPDH antibody; α-Fortilin, anti-fortilin antibody; α-PRX1, anti-PRX1 antibody. **G. PRX1 is less ubiquitinated in the liver of fortilin$^{Liver\text{-}Tg}$ mice than that of fortilin$^{Liver\text{-}WT}$ mice.** IP, immunoprecipitation; IB, immunoblot; α-Ubiquitin, anti-ubiquitin antibody; polyUb-PRX1, poly-ubiquitinated PRX1; Ub Index, PRX1 ubiquitination index. Transgenic overexpression of fortilin decreased the poly-ubiquitinated PRX1s in liver lysates as assessed by the PRX1 ubiquitination indices (fortilin$^{Liver\text{-}WT}$ vs. fortilin$^{Liver\text{-}Tg}$ mice = 1.0 vs. 0.36). See Materials and Methods for detail. **H. Ponceau S staining of proteins transferred to nitrocellulose membranes from the 2D gels.** Buffer, buffer-treated; AP, alkaline-phosphatase-treated; Ponceau S staining shows similar protein loading and transfer for each 2-D gel. After Ponceau S staining, the membranes were immunoblotted by anti-PRX1 antibody as depicted in **Fig. 4F**.

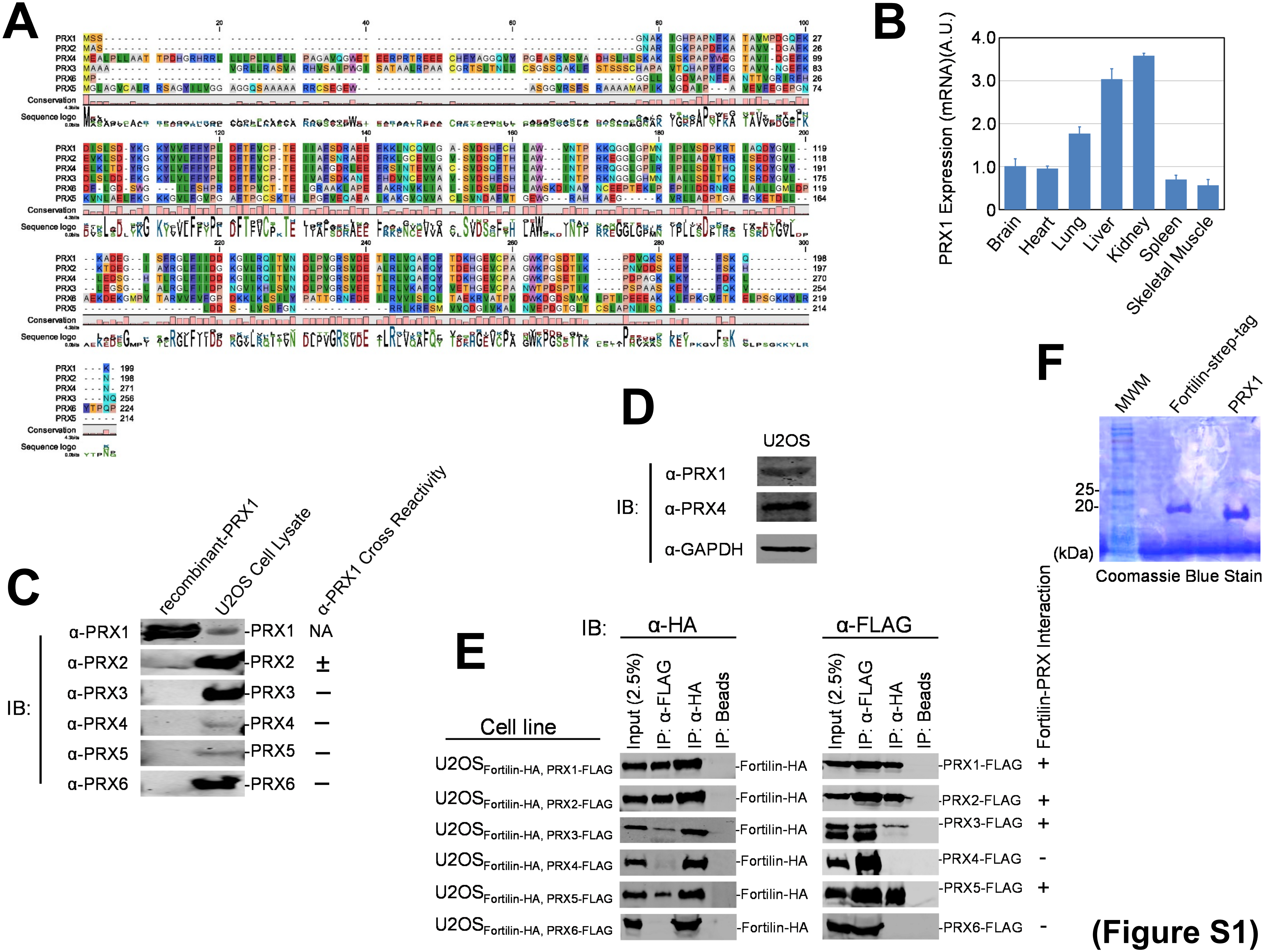
A
PRX1
PRX2
PRX4
PRX3
PRX6
PRX5
Conservation
Sequence logo
B
PRX1 Expression (mRNA)(A.U.)
4.0
3.0
2.0
1.0
0
Brain
Heart
Lung
Liver
Kidney
Spleen
Skeletal Muscle
C
recombinant-PRX1
U2OS Cell Lysate
α-PRX1 Cross Reactivity
IB:
α-PRX1
α-PRX2
α-PRX3
α-PRX4
α-PRX5
α-PRX6
-PRX1
-PRX2
-PRX3
-PRX4
-PRX5
-PRX6
NA
±
D
U2OS
IB:
α-PRX1
α-PRX4
α-GAPDH
E
IB:
α-HA
α-FLAG
Cell line
Input (2.5%)
IP: α-FLAG
IP: α-HA
IP: Beads
Fortilin-PRX Interaction
U2OS Fortilin-HA, PRX1-FLAG
U2OS Fortilin-HA, PRX2-FLAG
U2OS Fortilin-HA, PRX3-FLAG
U2OS Fortilin-HA, PRX4-FLAG
U2OS Fortilin-HA, PRX5-FLAG
U2OS Fortilin-HA, PRX6-FLAG
-Fortilin-HA
-PRX1-FLAG
-PRX2-FLAG
-PRX3-FLAG
-PRX4-FLAG
-PRX5-FLAG
-PRX6-FLAG
+
-
F
MWM
Fortilin-strep-tag
PRX1
25-
20-
(kDa)
Coomassie Blue Stain

(Figure S1)

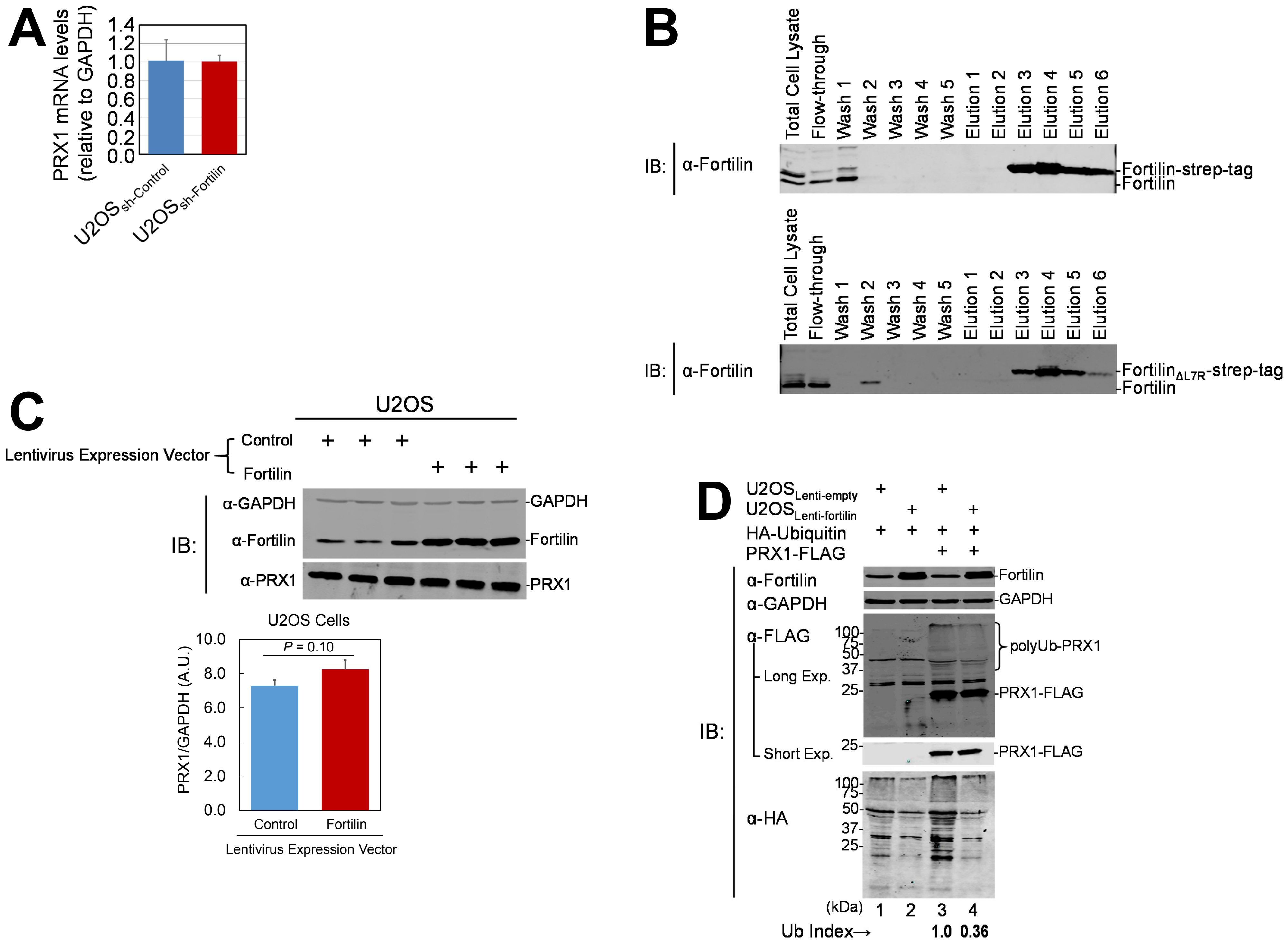

A
PRX1 mRNA levels (relative to GAPDH)
1.4
1.2
1.0
0.8
0.6
0.4
0.2
0.0
U2OS sh-Control
U2OS sh-Fortilin
B
Total Cell Lysate
Flow-through
Wash 1
Wash 2
Wash 3
Wash 4
Wash 5
Elution 1
Elution 2
Elution 3
Elution 4
Elution 5
Elution 6
IB: α-Fortilin
Fortilin-strep-tag
Fortilin
Fortilin ΔL7R-strep-tag
C
U2OS
Lentivirus Expression Vector
Control
Fortilin
IB:
α-GAPDH
α-Fortilin
α-PRX1
GAPDH
PRX1
U2OS Cells
P = 0.10
PRX1/GAPDH (A.U.)
10.0
8.0
6.0
4.0
2.0
0.0
Control
Fortilin
Lentivirus Expression Vector
D
U2OS Lenti-empty
U2OS Lenti-fortilin
HA-Ubiquitin
PRX1-FLAG
α-Fortilin
α-GAPDH
α-FLAG
Long Exp.
Short Exp.
α-HA
100
75
50
37
25
polyUb-PRX1
(kDa)
1
2
3
4
Ub Index→
1.0
0.36

(Figure. S2)

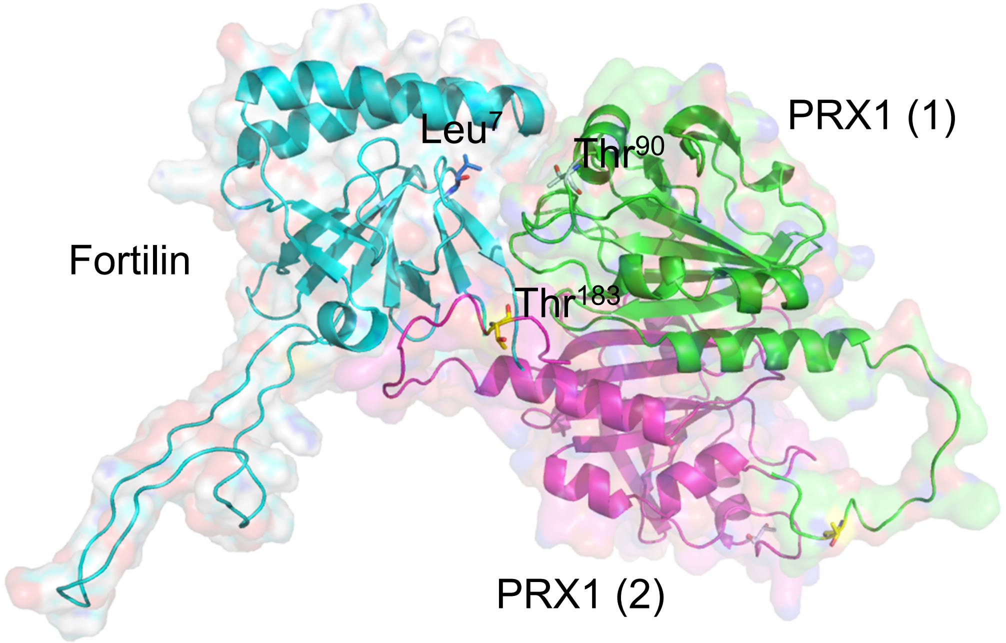

(Figure S3)

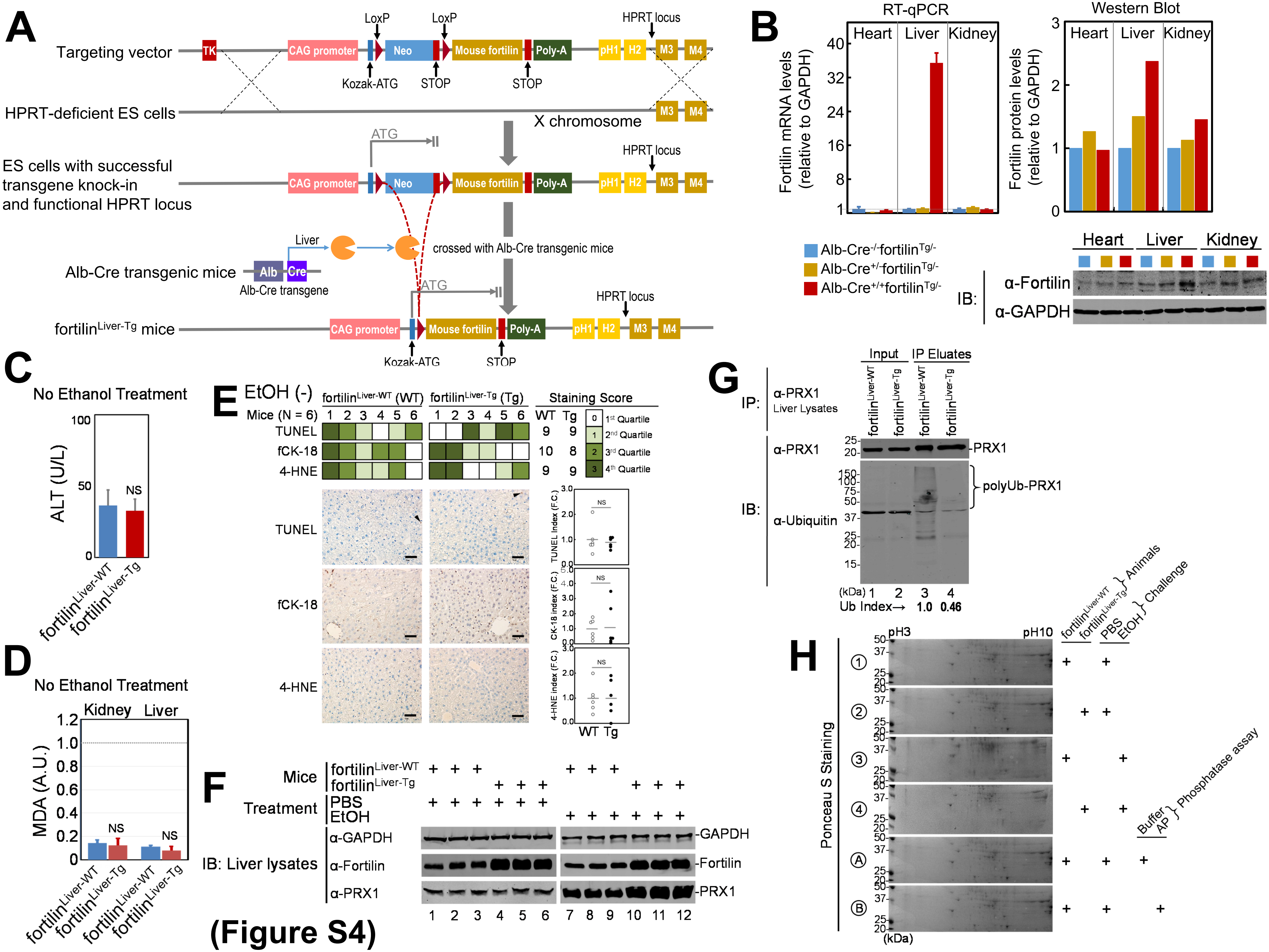

A
Targeting vector
TK
CAG promoter
LoxP
Neo
Mouse fortilin
Poly-A
pH1
H2
M3
M4
HPRT locus
Kozak-ATG
STOP
HPRT-deficient ES cells
X chromosome
ATG
ES cells with successful transgene knock-in and functional HPRT locus
Liver
crossed with Alb-Cre transgenic mice
Alb-Cre transgenic mice
Alb
Cre
Alb-Cre transgene
fortilinLiver-Tg mice
B
RT-qPCR
Western Blot
Heart
Liver
Kidney
Fortilin mRNA levels (relative to GAPDH)
Fortilin protein levels (relative to GAPDH)
Alb-Cre-/-fortilinTg/-
Alb-Cre+/-fortilinTg/-
Alb-Cre+/+fortilinTg/-
IB:
α-Fortilin
α-GAPDH
C
No Ethanol Treatment
ALT (U/L)
NS
fortilinLiver-WT
fortilinLiver-Tg
D
No Ethanol Treatment
Kidney
Liver
MDA (A.U.)
NS
E
EtOH (-)
fortilinLiver-WT (WT)
fortilinLiver-Tg (Tg)
Staining Score
Mice (N = 6)
TUNEL
fCK-18
4-HNE
1st Quartile
2nd Quartile
3rd Quartile
4th Quartile
TUNEL Index (F.C.)
CK-18 index (F.C.)
4-HNE index (F.C.)
WT
Tg
F
Mice
Treatment
PBS
EtOH
IB: Liver lysates
α-GAPDH
α-Fortilin
α-PRX1
-GAPDH
-Fortilin
-PRX1
G
IP:
α-PRX1
Liver Lysates
Input
IP Eluates
IB:
α-Ubiquitin
-PRX1
polyUb-PRX1
(kDa)
Ub Index→
1.0
0.46
H
pH3
pH10
Ponceau S Staining
Animals
Challenge
Buffer
AP
Phosphatase assay
(kDa)
(Figure S4)
